# Treatment outcomes with oral anti‐hyperglycaemic therapies in people with diabetes secondary to a pancreatic condition (type 3c diabetes): A population‐based cohort study

**DOI:** 10.1111/dom.16163

**Published:** 2025-01-06

**Authors:** Rhian Hopkins, Katherine G. Young, Nicholas J. Thomas, Angus G. Jones, Andrew T. Hattersley, Beverley M. Shields, John M. Dennis, Andrew P. McGovern, the MASTERMIND consortium

**Affiliations:** ^1^ Department of Clinical and Biomedical Sciences University of Exeter Medical School Exeter UK

**Keywords:** antidiabetic drug, effectiveness, glycaemic control, observational study, primary care, real‐world evidence

## Abstract

**Aims:**

To assess outcomes of oral anti‐hyperglycaemic therapies in people with diabetes secondary to a pancreatic condition (type 3c), where specific treatment guidance is limited.

**Materials and Methods:**

Using hospital‐linked UK primary care records (Clinical Practice Research Datalink; 2004–2020), we identified 7084 people with a pancreatic condition (acute pancreatitis, chronic pancreatitis, pancreatic cancer and haemochromatosis) preceding diabetes diagnosis (type 3c cohort), initiating oral glucose‐lowering therapy (metformin, sulphonylureas, SGLT2‐inhibitors, DPP4‐inhibitors or thiazolidinediones), and without concurrent insulin treatment. We stratified by pancreatic exocrine insufficiency [PEI] (*n* = 5917 without PEI, 1167 with PEI) and matched to 97 227 type 2 diabetes (T2D) controls. 12‐month HbA1c response and weight change and 6‐month treatment discontinuation were compared in type 3c versus T2D.

**Results:**

People with type 3c diabetes had substantial mean HbA1c reduction with oral therapies in those without PEI (12.2 [95%CI 12.0–12.4] mmol/mol) and with PEI (9.4 [8.9–10.0] mmol/mol). Compared to T2D controls, people with type 3c without PEI had similar mean HbA1c reduction (0.7 [0.4–1.0] mmol/mol difference) and odds of discontinuation (Odds ratio [OR] 1.08 [0.98–1.19]). In contrast, people with type 3c and PEI had lower mean HbA1c response (3.5 [2.9–4.1] mmol/mol lesser reduction) and greater discontinuation (OR 2.03 [1.73–2.36]) versus T2D controls. Weight change in type 3c was similar to T2D. Results were largely consistent across underlying pancreatic conditions and drug classes.

**Conclusions:**

Oral anti‐hyperglycaemic therapies are effective in people with type 3c diabetes and could provide an important component of glycaemic management. PEI could identify people with type 3c requiring closer monitoring of treatment response.

## 1 INTRODUCTION

Type 3c diabetes, also known as diabetes of the exocrine pancreas or pancreatogenic diabetes, results from damage to the pancreas and can be caused by a variety of pancreatic conditions. These include acute pancreatitis, chronic pancreatitis, pancreatic cancer, cystic fibrosis, haemochromatosis and surgical pancreatic resection.^1^, ^2^, ^3^, ^4^, ^5^, ^6^, ^7^ Type 3c diabetes comprises 5%–10% of people with diabetes in Western populations.^8^ The adult incidence is higher than that of type 1 diabetes^9^ and may also be increasing.^10^ In clinical practice, people with type 3c diabetes are most commonly classified as having type 2 diabetes but have worse glycaemic control and a greater requirement for insulin.^9^, ^11^

Guidelines for the management of type 3c diabetes are limited, and evidence to inform treatment decisions is inadequate.^8^, ^12^, ^13^ Treatment of hyperglycaemia in type 3c diabetes is not clearly differentiated from that for type 1 and type 2 diabetes,^6^ with current recommendations limited to considering early insulin initiation and avoidance of incretin‐based therapies due to their potential association with pancreatitis.^12^ There is a lack of studies providing evidence on whether oral diabetes therapies are effective and safe in the management of hyperglycaemia in type 3c diabetes.^6^, ^7^, ^14^ There are also no dedicated randomized controlled trials of treatments in type 3c diabetes, and people with this type of diabetes are usually excluded from major diabetes drug trials.^2^, ^8^, ^15^ Due to misclassification of diabetes type and a lack of treatment guidelines, a substantial proportion of people with this type of diabetes may be receiving glucose‐lowering therapies that are sub‐optimal.^15^, ^16^

Oral glucose‐lowering therapies could potentially be less effective in type 3c diabetes, and their effectiveness likely varies by the degree of pancreatic damage. However, treatment response in these patients has not been systematically assessed. Type 3c diabetes is a very heterogenous condition,^7^, ^17^ and therefore the different underlying causes could also potentially result in different treatment responses. In addition to the endocrine dysfunction that results in diabetes, the exocrine functions of the pancreas can be damaged resulting in pancreatic exocrine insufficiency (PEI).^7^, ^9^ This arises when the production of pancreatic enzymes that typically digest food and absorb nutrients is impaired^6^ and is a key feature of this type of diabetes.^12^ We hypothesised that PEI could be a marker of the severity of pancreatic damage and could therefore predict effectiveness of oral therapies.

We aimed to assess treatment outcomes to oral anti‐hyperglycaemic therapies in people with diabetes secondary to a pancreatic condition (type 3c diabetes) and compare them to those with type 2 diabetes.

## 2 MATERIALS AND METHODS

### 2.1 Data sources

UK population‐based primary care data from the Clinical Practice Research Datalink (CPRD), a large database of routinely collected medical records, covering demographics, diagnoses, prescriptions and test results from over 2000 primary care practices.^18^, ^19^ CPRD data were linked to national Hospital Episode Statistics (HES) and Index of Multiple Deprivation data (national measure of deprivation).^20^

### 2.2 Study participants

We extracted primary care records and linked hospital records on all people with a diabetes diagnosis from 2004 to 2020. People with diabetes were identified from the primary care records using clinical codes (underlying data is coded using Read and SNOMED CT coding systems, CPRD specific medical codes used to identify diabetes available from: https://github.com/Exeter-Diabetes/CPRD-Codelists). We defined diabetes diagnosis dates as the date of the earliest diabetes clinical code, prescription for a glucose‐lowering medication or HbA1c ≥48 mmol/mol.

#### 2.2.1 Type 3c diabetes

We identified people with a diagnosis of any type of diabetes who had a pancreatic condition prior to or up to 30 days after their diabetes diagnosis date (type 3c diabetes cohort). We defined pancreatic conditions as a record of acute pancreatitis, chronic pancreatitis, pancreatic cancer or haemochromatosis in the primary or secondary care records. We identified and excluded from our type 3c diabetes cohort anyone with surgical pancreatic resection only or cystic fibrosis prior to diabetes diagnosis as surgical pancreatic resection included both partial resection and total pancreatectomy which were difficult to differentiate between, and the management for cystic fibrosis‐related diabetes is already well defined.^21^

We stratified our type 3c diabetes cohort by the presence/absence of PEI in order to evaluate whether this feature impacts treatment response. We defined PEI as a PEI diagnosis code, or a faecal elastase‐1 (FE1) test result less than 200 ug/g,^22^ or a prescription for pancreatic enzyme replacement therapy (PERT), prior to diabetes diagnosis.

#### 2.2.2 Type 2 diabetes

As a comparator group, we identified people with type 2 diabetes (T2D) and no record of a prior pancreatic condition. We classified T2D using a published algorithm based on diabetes clinical code counts, glucose‐lowering medication prescriptions and diagnosis age. This classification approach has been validated against the use of genetic and biochemical markers of diabetes type.^23^ Other types of diabetes were excluded (Figure S1).

### 2.3 Treatment outcome cohort

For both type 3c diabetes and T2D, treatment outcome cohorts were defined as people initiating a major oral glucose‐lowering therapy class (metformin, sulphonylureas, thiazolidinediones, DPP4‐inhibitors or SGLT2‐inhibitors) for the first time, with the index date set to the date of initiation of that therapy class. GLP‐1 receptor agonists were not evaluated as they were initiated by only 258 people with type 3c diabetes. If an individual initiated more than one therapy class over the study period, they were eligible for inclusion in evaluation of each initiated drug class. We excluded people treated with concurrent insulin therapy, those with T2D who developed a pancreatic condition before drug initiation or with T2D and a record of PEI at any time and those with type 3c diabetes with a record of PEI only after drug initiation (Figure S1).

People with type 3c diabetes were then matched to up to 10 T2D controls initiating the same drug class, without replacement, using a combination of exact matching by sex, ethnicity (White, south Asian, Black, Other, Mixed and unknown), deprivation (Index of Multiple Deprivation quintiles: 1 (least deprived) – 5 (most deprived)), age (10‐year bands), calendar year (5‐year bands) and the number of previously initiated anti‐hyperglycaemic drug classes (0,1,2+) and nearest‐neighbour matching by continuous age and calendar year of drug initiation (Figures S1 and S2).

### 2.4 Outcomes and covariates

We defined 3 treatment response outcomes: HbA1c response, early discontinuation and weight change. HbA1c response and weight change were defined as the change from baseline 12 months after drug initiation (the closest measure to 12 months after initiation within 3–15 months) on unchanged therapy (no addition or cessation of other glucose‐lowering medications and continued prescription of the drug of interest). Early treatment discontinuation was defined as the discontinuation of a therapy within 6 months of initiation. A gap of over 6 months in prescriptions was used to indicate a drug being stopped, and the availability of at least 3 months follow‐up time after discontinuation was required to confirm the drug was discontinued. Treatment response outcome definitions are consistent with previous studies^24^ and are also given in Table S1. Secondary outcomes were time to insulin initiation and time to oral agent initiation.

We extracted baseline sociodemographic and clinical features comprising: sex, age, ethnicity, deprivation, baseline HbA1c, baseline BMI, baseline weight, number of other glucose‐lowering therapies currently being taken, alcohol consumption, duration of diabetes, microvascular complications (diabetic nephropathy, retinopathy, neuropathy) and macrovascular complications (heart failure, atherosclerotic cardiovascular disease and chronic kidney disease). Further details are given in Table S2.

### 2.5 Statistical analysis

In people with type 3c diabetes and T2D, with GP data covering diabetes diagnosis, we assessed clinical and socio‐demographic characteristics at diagnosis. We evaluated time to insulin initiation and time to oral agents up to 3 years from diabetes diagnosis by PEI status and by underlying pancreatic condition, using Kaplan–Meier survival curves and unadjusted Cox proportional hazards models, censoring for death, the end of an individual's GP records or end of the study period.

When assessing oral therapy outcomes, our exposure of interest was diabetes type: type 3c diabetes without PEI, type 3c diabetes with PEI or T2D. We evaluated HbA1c response and weight changes using linear regression, adjusted for baseline measures. Discontinuation was evaluated using logistic regression, adjusted for baseline HbA1c. All models were adjusted for the number of other glucose‐lowering therapies being taken.

Baseline HbA1c and weight differ by drug class.^25^ To allow comparison of treatment outcomes by drug class, adjusted estimates of HbA1c response and discontinuation were standardised to the mean baseline HbA1c of 73 mmol/mol and weight change to the mean baseline weight of 93 kg. All outcomes were standardised for 1 other glucose‐lowering therapy currently being taken as most of the studied drug classes are second‐line treatments (0 if the drug class was metformin as this is standard first‐line treatment^25^).

Analysis was conducted overall, stratified by underlying pancreatic condition, and by drug class (with the exception of weight change as different drug classes are known to have opposite effects on weight change^25^). For all models, T2D controls were weighted as the inverse of the number of controls matched to an individual with type 3c diabetes. People with missing covariate or outcome data were excluded from each analysis.

### 2.6 Sensitivity analysis

Alcoholism is a common cause of pancreatitis and also negatively affects medication compliance in chronic conditions.^26^ To assess the potential impact of this, we repeated the overall treatment response analysis excluding people with a history of excess alcohol usage. In order to assess if our results were sensitive to our PEI definition, we also repeated overall analysis restricting the PEI subgroup definition to require a PERT prescription in the 6 months prior to drug initiation. To assess our definition of diabetes secondary to acute pancreatitis, where it is likely we will have included some people with T2D unrelated to their prior pancreatic condition, we repeated overall analysis in this subgroup in people with a record of acute pancreatitis within the 5 years prior to diabetes diagnosis.

Further information on study methods is available online (code lists: https://github.com/Exeter-Diabetes/CPRD-Codelists, defining cohorts: https://github.com/Exeter-Diabetes/CPRD-Cohort-scripts/, analysis: https://github.com/Exeter-Diabetes/CPRD-Rhian-T3cTreatment-Scripts). Statistical analysis was performed using R version 4.3.0, and RECORD reporting guidelines were followed.^27^

## 3 RESULTS

### 3.1 Type 3c diabetes clinical characteristics at diagnosis

10 318 people with type 3c diabetes (8800 without PEI and 1518 with PEI) and 524 084 people with T2D had GP data covering diabetes diagnosis. Those with type 3c diabetes, particularly those with PEI, were more commonly male, of White ethnicity, and had greater deprivation, compared to those with T2D (Table 1). Those with type 3c diabetes and PEI also had a lower BMI at diagnosis. HbA1c was largely similar between groups. People with type 3c diabetes and PEI most commonly had prior chronic pancreatitis (72.1%), followed by prior pancreatic cancer (19.2%), whereas the majority of those with type 3c diabetes without PEI had prior acute pancreatitis (59.0%).

**TABLE 1:** Baseline clinical and socio‐demographic characteristics at diabetes diagnosis and treatment after diagnosis in individuals with type 3c diabetes and type 2 diabetes controls.

	Type 2 diabetes	Type 3c diabetes		
		Overall	PEI	No PEI
*n*	524 084	10 318	1518	8800
Sex, *n* (%)				
Male	295 514 (56.4)	6071 (58.8)	965 (63.6)	5106 (58.0)
Female	228 570 (43.6)	4247 (41.2)	553 (36.4)	3694 (42.0)
Age, years				
Median [IQR]	60.90 [51.00, 70.67]	60.30 [49.60, 70.71]	58.38 [48.28, 67.70]	60.74 [49.82, 71.35]
Ethnicity, *n* (%)				
White	421 553 (82.7)	9265 (90.6)	1432 (94.7)	7833 (89.9)
South Asian	51 054 (10.0)	536 (5.2)	40 (2.6)	496 (5.7)
Black	25 492 (5.0)	249 (2.4)	27 (1.8)	222 (2.5)
Other	7142 (1.4)	91 (0.9)	7 (0.5)	84 (1.0)
Mixed	4670 (0.9)	80 (0.8)	6 (0.4)	74 (0.8)
Index of multiple deprivation quintile, *n* (%)				
1 (least deprived)	93 211 (18.0)	1790 (17.5)	253 (16.8)	1537 (17.7)
2	97 264 (18.8)	1777 (17.4)	277 (18.4)	1500 (17.2)
3	100 360 (19.4)	1954 (19.2)	279 (18.6)	1675 (19.3)
4	109 983 (21.2)	2189 (21.5)	308 (20.5)	1881 (21.6)
5 (most deprived)	117 611 (22.7)	2492 (24.4)	387 (25.7)	2105 (24.2)
HbA1c, mmol/mol				
Median [IQR]	53.00 [48.81, 67.10]	53.00 [49.00, 72.67]	54.04 [49.00, 78.44]	53.00 [49.00, 71.79]
BMI, kg/m^2^				
Median [IQR]	31.60 [27.90, 36.10]	29.64 [25.40, 34.20]	24.80 [21.30, 28.83]	30.30 [26.30, 34.90]
3c subtype, *n* (%)				
Acute pancreatitis only	NA	5322 (51.6)	129 (8.5)	5193 (59.0)
Chronic pancreatitis	NA	3623 (35.1)	1095 (72.1)	2528 (28.7)
Haemochromatosis	NA	681 (6.6)	3 (0.2)	678 (7.7)
Pancreatic cancer	NA	692 (6.7)	291 (19.2)	401 (4.6)
Treatment after diagnosis				
Insulin within 1 year, % (95% CI)	2.0 [1.9,2.0]	18.4 [17.7,19.2]	32.8 [30.4,35.2]	15.9 [15.2,16.7]
Insulin within 3 years, % (95% CI)	3.5 [3.4,3.5]	23.7 [22.9,24.6]	43.7 [41.1,46.2]	20.3 [19.5,21.2]
Oral agent within 1 year, % (95% CI)	49.8 [49.7,49.9]	51.0 [50.0,51.9]	48.9 [46.4,51.4]	51.3 [50.3,52.3]
Oral agent within 3 years, % (95% CI)	64.8 [64.7,65.0]	65.1 [64.1,66.0]	60.4 [57.8,62.9]	65.9 [64.8,66.9]

### 3.2 Treatment after diagnosis

Early insulin initiation was more common in people diagnosed with type 3c diabetes compared to people with T2D, most marked in those with PEI (PEI: 43.7% insulin treated at 3 years; hazard ratio [HR] 17.27 [95% CI 15.96–18.69], without PEI: 20.3% insulin treated at 3 years; HR 6.79 [95% CI 6.46–7.13] [Table 1, Figure S3]). There was also heterogeneity by underlying pancreatic condition, with the greatest early insulin initiation in pancreatic cancer (HR 19.48 [95% CI 17.37–21.84]) and chronic pancreatitis subtypes (HR 13.82 [95% CI 13.06–14.62]) (Figure S4). The proportion of people initiating oral agents following diabetes diagnosis was largely similar between people with type 3c diabetes and those with T2D, although there was less oral therapy initiation at 3 years in those with PEI (HR 0.93 [95% CI 0.87–1.00]) and those with pancreatic cancer (HR 0.83 [95% CI 0.75–0.92]) (Figures S5 and S6).

### 3.3 Overall oral therapy outcomes

7084 people with type 3c diabetes initiated an oral glucose‐lowering therapy (5917 without PEI and 1167 with PEI) and were matched to 97 227 T2D controls (Figure S1, baseline characteristics in Table S3).

**FIGURE 1:**
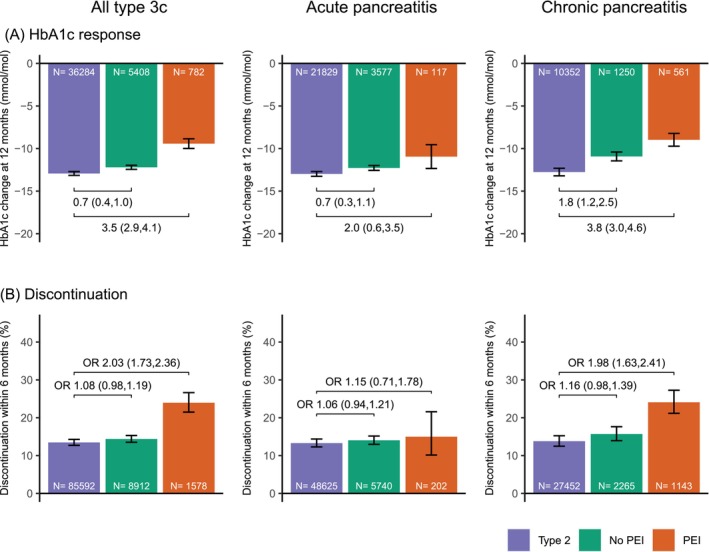
(A) Mean HbA1c response and (B) proportion of early treatment discontinuation, in individuals with type 3c diabetes and PEI (orange), type 3c diabetes without PEI (green) and matched type 2 diabetes controls (blue/purple) initiating an oral glucose‐lowering therapy, for the full type 3c cohort, those with type 3c diabetes following acute pancreatitis and those with type 3c diabetes following chronic pancreatitis. Contrasts represent estimated differences between groups with 95% confidence intervals for HbA1c change in mmol/mol and odds ratios with 95% confidence intervals for discontinuation. Models were adjusted for baseline HbA1c and number of other glucose‐lowering therapies being taken.

In the type 3c diabetes treatment outcome cohort, a substantial mean HbA1c response with oral therapy was observed: 12.2 mmol/mol (95% CI 12.0–12.4) in people without PEI and 9.4 mmol/mol (95% CI 8.9–10.0) in people with PEI (Figure 1A). Compared to the T2D controls, HbA1c response was largely similar in those without PEI (0.7 [95% CI 0.4–1.0] mmol/mol lesser mean HbA1c response) but lower in those with PEI (3.5 [95% CI 2.9–4.1] mmol/mol lesser response).

Early discontinuation of oral therapy within 6 months was 11.2% in the T2D controls, 12.8% in those with type 3c diabetes without PEI, and 20.0% in those with PEI. Compared to T2D controls, the odds of discontinuation in people with type 3c diabetes were similar in those without PEI (odds ratio [OR] 1.08 [95% CI 0.98–1.19]) but higher in those with PEI (OR 2.03 [95% CI 1.73–2.36]; Figure 1B).

### 3.4 Oral therapy outcomes by Type 3c diabetes subtype

There was a similar pattern of treatment outcomes when assessing by underlying pancreatic condition subtype, with a modestly reduced glycaemic response in those with type 3c diabetes for both chronic pancreatitis and acute pancreatitis subtypes compared to T2D controls, most marked in those with PEI (Figure 1A). Discontinuation was higher versus T2D controls in the chronic pancreatitis subgroup with PEI, with no evidence of a difference for other subgroups (Figure 1B). In those with pancreatic cancer and PEI, there was reduced glycaemic response (3.1 [95% CI 1.3–4.9] mmol/mol lesser HbA1c reduction) and increased discontinuation compared to T2D controls (OR 2.60 [95% CI 1.60–4.25]), with no difference in either outcome for those without PEI (Figure S7). In the haemochromatosis subtype, only 4 people had PEI (treatment outcome differences therefore not analysed) and in those without PEI glycaemic response and discontinuation were similar to the T2D controls (Figure S8).

### 3.5 Oral therapy outcomes by drug class

**FIGURE 2:**
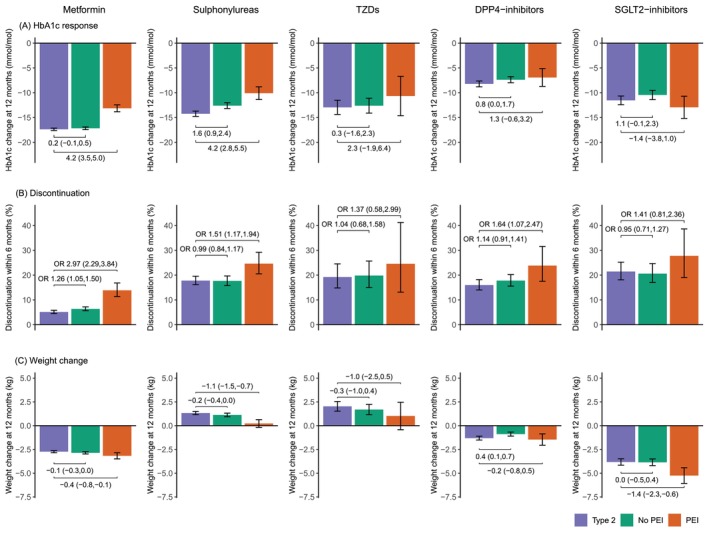
(A) Mean HbA1c response, (B) proportion of early treatment discontinuation and (C) mean weight change, in all individuals with type 3c diabetes and PEI (orange), type 3c diabetes without PEI (green) and matched type 2 diabetes controls (blue/purple) initiating an oral glucose‐lowering therapy, for different classes of oral glucose‐lowering therapy: Metformin, sulphonylureas, thiazolidinediones [TZDs], DPP4‐inhibitors and SGLT2‐inhibitors, respectively. Contrasts represent estimated differences between groups with 95% confidence intervals for HbA1c change in mmol/mol and weight change in kg and odds ratios with 95% confidence intervals for discontinuation. Models were adjusted for baseline values and number of other glucose‐lowering therapies being taken. Numbers in each subgroup are given in Table S4.

People with type 3c diabetes showed a substantial HbA1c response across all drug classes when studied separately. Those without PEI had a slightly lesser response versus T2D controls for sulfonylureas only (1.6 [95% CI 0.9–2.4] mmol/mol lesser response; Figure 2A). In those with PEI, mean response was lower compared to T2D controls for metformin and sulphonylureas (both 4.2 mmol/mol lesser response; Figure 2A). There was no evidence of a reduced response on SGLT2‐inhibitors or thiazolidinediones in either group.

Short‐term discontinuation in people with type 3c diabetes without PEI was broadly similar to T2D across all drug classes, with the exception of metformin (OR 1.26 [95% CI 1.05–1.50]). In people with type 3c diabetes and PEI, discontinuation was greater versus T2D controls across all drug classes, with the most marked increase seen for metformin (OR 2.97 [95% CI 2.29–3.84]; Figure 2B).

Weight change varied by drug class but was largely similar between people with type 3c diabetes and T2D controls (Figure 2C). The exception was for people with type 3c diabetes and PEI, who had greater mean weight loss on SGLT2‐inhibitors compared to T2D controls (1.4 [95% CI 0.6–2.3] kg greater weight loss) and lesser mean weight gain on sulphonylureas (1.1 [95% CI 0.7–1.5] kg lesser weight gain). Tables S4–S8 report full numerical unadjusted and adjusted treatment outcome results, stratified by both underlying pancreatic condition and drug class.

### 3.6 Sensitivity analyses

Treatment outcomes were largely consistent with the primary analysis when (1) including only people whose alcohol consumption was within recommended limits or who did not consume alcohol at drug initiation (Figure S9), (2) restricting the cohort of people with type 3c diabetes and PEI to those with a PERT prescription in the 6 months prior to drug initiation (Figure S10) and (3) restricting the criteria of diabetes following acute pancreatitis to those with a record of acute pancreatitis within 5 years prior to diabetes diagnosis (Figure S11).

## 4 DISCUSSION

In this large UK observational study, we found that people with type 3c diabetes had a substantial HbA1c reduction to five major oral anti‐hyperglycaemic drug classes, largely comparable to those with T2D. We also identified clinically relevant heterogeneity within the broad type 3c diabetes phenotype. In particular, PEI, a potential marker of the severity of pancreatic damage, was identified as a novel determinant of treatment outcomes on oral therapy; those with PEI had a modestly reduced glycaemic response and increased risk of early treatment discontinuation compared to people with T2D. In contrast, those without PEI, who make up the majority of our type 3c diabetes cohort, had an only slightly lesser glycaemic response (<1 mmol/mol average difference) and similar risk of discontinuation compared to T2D. Findings were broadly consistent across the evaluated drug classes (metformin, sulphonylureas, DPP4‐inhibitors, thiazolidinediones and SGLT2‐inhibitors) and suggest that PEI represents a simple clinical marker that may be a more important determinant of treatment outcomes than the underlying pancreatic condition in type 3c diabetes.

Glycaemic response in those with PEI was most attenuated with metformin and sulphonylureas (4.2 mmol/mol lesser response than T2D), although both therapies were still effective. This may be because these medication classes influence the secretion or action of endogenous insulin, processes potentially more adversely affected in type 3c diabetes than T2D, particularly in those with PEI where pancreatic damage may be more severe. A reduced response was not seen for SGLT2 inhibitors, which work independently of endogenous insulin.^15^ Metformin discontinuation was the most different between type 3c diabetes and T2D with threefold higher odds of discontinuation in those with type 3c diabetes and PEI compared to T2D. This may relate to the known gastrointestinal side effects of metformin, given PEI causes similar symptoms, and could mean the therapy may not be well‐tolerated in some people with type 3c diabetes.^15^, ^28^ The impact of oral medications on weight change was mostly similar in type 3c diabetes and T2D, although small differences for sulphonylureas and SGLT2‐inhibitors in those with PEI were observed, these are unlikely to be clinically significant.

As previously observed in smaller datasets, we observed greater early insulin initiation in people with type 3c diabetes compared to T2D,^9^, ^16^, ^29^, ^30^ particularly in people with chronic pancreatitis or pancreatic cancer. This may be due to more impaired endogenous insulin production as a result of pancreatic damage,^2^, ^7^ but could also reflect the tendency of clinicians to prescribe insulin when type 3c diabetes is suspected given the uncertain effectiveness of oral medications in this patient group.

### 4.1 Strengths and limitations

To our knowledge, this study is the first to assess short‐term outcomes to oral therapies in type 3c diabetes, and whether outcomes vary by PEI status, drug class, and underlying pancreatic condition. A strength of this study is the large size of the dataset, enabling analysis of a much larger cohort of people with type 3c diabetes than many defined using other observational datasets.^9^, ^16^, ^29^, ^30^, ^31^ However, we acknowledge that when stratifying by underlying pancreatic condition and drug class, low numbers still limit these analyses. We were unable to include GLP1 receptor agonists in our study or to assess responses to oral therapies in people concurrently treated with insulin due to low numbers. The lack of a standardized approach to identify type 3c diabetes in routine clinical data is a limitation, and many people with type 3c diabetes will have been labelled with T2D in practice.^9^ In the absence of universally accepted diagnostic criteria, we adopted a similar pragmatic approach to previous observational studies of type 3c diabetes and classified people based on prior records of a pancreatic condition.^9^, ^16^, ^29^, ^31^ As a result, there will likely be people in our type 3c diabetes cohort who have T2D that may have occurred regardless of their prior pancreatic condition, but this is difficult to determine in our dataset, or indeed in clinical practice. This issue is potentially most likely to occur in our acute pancreatitis subgroup, and so we performed a sensitivity analysis restricting our type 3c diabetes definition to acute pancreatitis close to the date of diabetes diagnosis, which reassuringly showed results consistent with the primary analysis. We also excluded the small number of individuals (*n* = 440) with PEI first recorded after drug initiation from our study cohort. The rationale for this is that PEI is not systematically tested in UK clinical practice, meaning for many individuals in this group PEI is likely to have existed for a considerable time before it was identified. Excluding this group therefore helped to avoid contamination of the type 3c diabetes group without PEI with people who may have had undiagnosed PEI. CPRD data are collected as part of routine care and is widely representative of the general population with many ethnic groups represented.^18^ However, challenges of routine records include the lack of data on duration of prescriptions and medication adherence to precisely identify treatment discontinuation, as well as missing data. A limitation of our study is the exclusion of individuals with missing treatment outcomes, although our previous work using the same dataset demonstrates that missing data does not meaningfully alter treatment effect estimates with oral glucose‐lowering therapy.^32^ A key strength of this study was the use of both primary care records and hospital admission data to identify records of prior pancreatic conditions in order to identify type 3c diabetes phenotypes as robustly as possible.

### 4.2 Implications

People with type 3c diabetes have commonly been excluded from major diabetes drug trials,^2^, ^8^, ^15^ and no previous studies have assessed whether and which oral glucose‐lowering therapies are effective in treating this group. We have demonstrated that oral glucose‐lowering therapies are effective in non‐insulin treated people with type 3c diabetes, and for the majority of this group there is no meaningful difference in glycaemic response or tolerability to those with T2D. This suggests that oral therapies could provide an important component of glycaemic management in this group. While oral glucose‐lowering therapies remained effective in those with PEI, there was a modest reduction in effectiveness and likely reduction in tolerability for most drug classes, in comparison to T2D, suggesting people with type 3c diabetes and PEI may benefit from closer monitoring when making a shared decision to initiate oral diabetes treatment. Future studies should evaluate other patient important outcomes, including the safety of these therapies in people with type 3c diabetes, and address concerns surrounding drug‐specific side effects, for example the risk of hypoglycaemia with sulphonylureas,^15^ and the potential association of DPP4‐inhibitors and increased risk of pancreatitis.^12^ Now that we have established that oral therapies are effective in type 3c diabetes, an important question for future research is to examine the relative effectiveness of different therapy classes within this group.

## 5 CONCLUSION

In this large UK population‐based study, we observed a substantial HbA1c response to five major oral anti‐hyperglycaemic therapies in people with type 3c diabetes. By demonstrating that these agents are effective at treating hyperglycaemia in people with type 3c diabetes, our findings suggest oral therapy could provide an important component of glycaemic management. However, PEI was associated with modestly reduced glycaemic response and likely reduced tolerability, and therefore people with PEI may benefit from closer monitoring after initiating oral therapy.

## AUTHOR CONTRIBUTIONS

The study concept and design were developed by A.P.M., J.M.D. and R.H., K.G.Y., and R.H. prepared the data for analysis. R.H. undertook the analysis, with support from J.M.D., B.M.S., and A.P.M., A.P.M., A.G.J., N.J.T., and A.T.H., provided clinical insight. All authors provided support for the interpretation of results, critically revised the manuscript and saw and approved the final article. A.P.M. and J.M.D. attest that all listed authors meet authorship criteria that no others meeting the criteria have been omitted. A.P.M. and J.M.D. are responsible for the decision to submit for publication, are the guarantors of this work and, as such, had full access to all the data in the study and takes responsibility for the integrity of the data and the accuracy of the data analysis.

## FUNDING INFORMATION

This research was funded by the Medical Research Council (UK) (MR/N00633X/1). The funder had no role in any part of the study or in any decision about publication. RH and KGY are supported by Research England's Expanding Excellence in England (E3) fund. BMS and ATH are supported by the NIHR Exeter Clinical Research Facility. JMD is supported by a Wellcome Trust Early Career award (227 070/Z/23/Z).

## CONFLICT OF INTEREST STATEMENT

APM received prior research funding from Eli Lilly and Company, Pfizer and AstraZeneca outside of the submitted work. All other authors declare no other relationships or activities that could appear to have influenced the submitted work.

### PEER REVIEW

The peer review history for this article is available at https://www.webofscience.com/api/gateway/wos/peer‐review/10.1111/dom.16163.

## ETHICS STATEMENT

The study protocol was approved by the CPRD Independent Scientific Advisory Committee (eRAP protocol numbers: 22_002000).

## PRIOR PRESENTATION

Oral presentation of preliminary results from this study has been given at the Diabetes UK Professional Conference 2024 and the European Association for the Study of Diabetes (EASD) Annual Meeting 2023.

## PATIENT AND PUBLIC INVOLVEMENT

The findings of this study were discussed with an individual with type 3c diabetes, which informed the interpretation of results. There was no patient or public involvement in the study design or analysis.

## RIGHTS RETENTION

For the purpose of open access, the author has applied a Creative Commons Attribution (CC BY) licence to any Author Accepted Manuscript version arising from this submission.

## Supporting information


Data S1:

File S1:

## Data Availability

No additional data are available from the authors although CPRD data are available by application to CPRD Independent Scientific Advisory Committee.
