# Supplementary material for: Treatment outcomes with oral anti‐hyperglycaemic therapies in people with diabetes secondary to a pancreatic condition (type 3c diabetes): A population‐based cohort study
Source: Diabetes Obes Metab. 2025 Jan 6;27(3):1544–53. doi: 10.1111/dom.16163 (PMC11802396; doi:10.1111/dom.16163)

Supplementary material: Treatment outcomes with oral anti-hyperglycaemic therapies in people with diabetes secondary to a pancreatic condition (type 3c diabetes): A population-based cohort study

Table of Contents

Page	
**2**	Supplementary Figure 1. Flow diagram of inclusion/exclusion criteria for the study treatment response cohort of individuals with type 3c and type 2 diabetes.
**3**	Supplementary Figure 2. Initiations of each drug class before and after matching.
**4**	Supplementary Table 1. Definitions of treatment outcomes.
**5**	Supplementary Table 2. Definitions of covariates.
**6**	Supplementary Figure 3. Kaplan-Meier cumulative incidence curve of time to initiation of insulin within 3 years of diabetes diagnosis for individuals with type 3c diabetes and PEI prior to diabetes diagnosis, type 3c diabetes without PEI prior to diagnosis, and individuals with type 2 diabetes.
**7**	Supplementary Figure 4. Kaplan-Meier cumulative incidence curve of time to initiation of insulin within 3 years of diabetes diagnosis for individuals with type 3c diabetes, stratified by subtype, and individuals with type 2 diabetes.
**8**	Supplementary Figure 5. Kaplan-Meier cumulative incidence curve of time to initiation of oral glucose-lowering therapy within 3 years of diabetes diagnosis for individuals with type 3c diabetes and PEI prior to diabetes diagnosis, type 3c diabetes without PEI prior to diagnosis, and individuals with type 2 diabetes.
**9**	Supplementary Figure 6. Kaplan-Meier cumulative incidence curve of time to initiation of oral glucose-lowering therapy within 3 years of diabetes diagnosis for individuals with type 3c diabetes, stratified by subtype, and individuals with type 2 diabetes.
**10-11**	Supplementary Table 3. Baseline characteristics of matched cohort of individuals with type 3c diabetes and type 2 controls initiating a major glucose-lowering therapy class (metformin, sulphonylureas, thiazolidinediones [TZDs], SGLT2-inhibitors, DPP4-inhibitors).
**12**	Supplementary Figure 7. A) Mean HbA1c response and B) Proportion of early treatment discontinuation, in individuals with type 3c diabetes following pancreatic cancer with PEI (orange) and without PEI (green), and matched type 2 controls (blue/purple) initiating an oral glucose lowering therapy.
**13**	Supplementary Figure 8. A) Mean HbA1c response and B) Proportion of early treatment discontinuation, in individuals with type 3c diabetes following haemochromatosis without PEI (green), and matched type 2 controls (blue/purple) initiating an oral glucose lowering therapy.
**14**	Supplementary Table 4. Treatment response outcomes by drug class in all individuals with type 3c diabetes and type 2 controls.
**15**	Supplementary Table 5. Treatment response outcomes by drug class in individuals with type 3c diabetes following acute pancreatitis and type 2 controls.
**16**	Supplementary Table 6. Treatment response outcomes by drug class in individuals with type 3c diabetes following chronic pancreatitis and type 2 controls.
**17**	Supplementary Table 7. Treatment response outcomes by drug class in individuals with type 3c diabetes following pancreatic cancer and type 2 controls.
**18**	Supplementary Table 8. Treatment response outcomes by drug class in individuals with type 3c diabetes following haemochromatosis and type 2 controls.
**19**	Supplementary Figure 9. Sensitivity analysis restricted to individuals whose alcohol consumption was within recommended limits or who did not consume alcohol.
**20**	Supplementary Figure 10. Sensitivity analysis restricting the criteria of the PEI subgroup to those with a PERT prescription within the 6 months prior to drug initiation.
**21**	Supplementary Figure 11. Sensitivity analysis restricting the criteria of diabetes following acute pancreatitis to those with a record of acute pancreatitis within the 5 years prior to diabetes diagnosis.

Supplementary Figure 1. Flow diagram of inclusion/exclusion criteria for the study treatment response cohort of individuals with type 3c and type 2 diabetes.

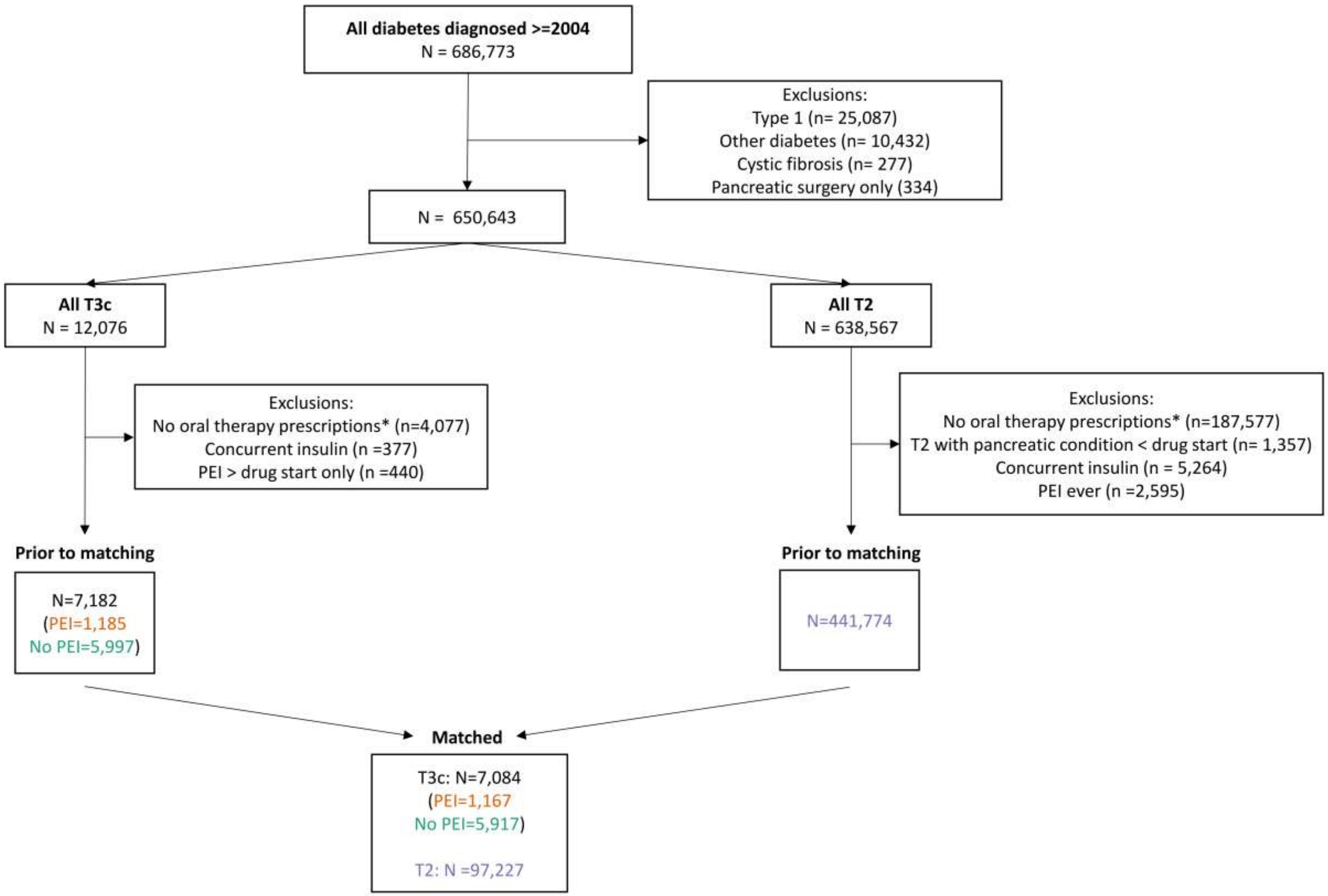

* Metformin, Sulphonylureas, TZDs, SGLT2- inhibitors, or DPP4-inhibitors

Supplementary Figure 2. Initiations of each drug class before and after matching.

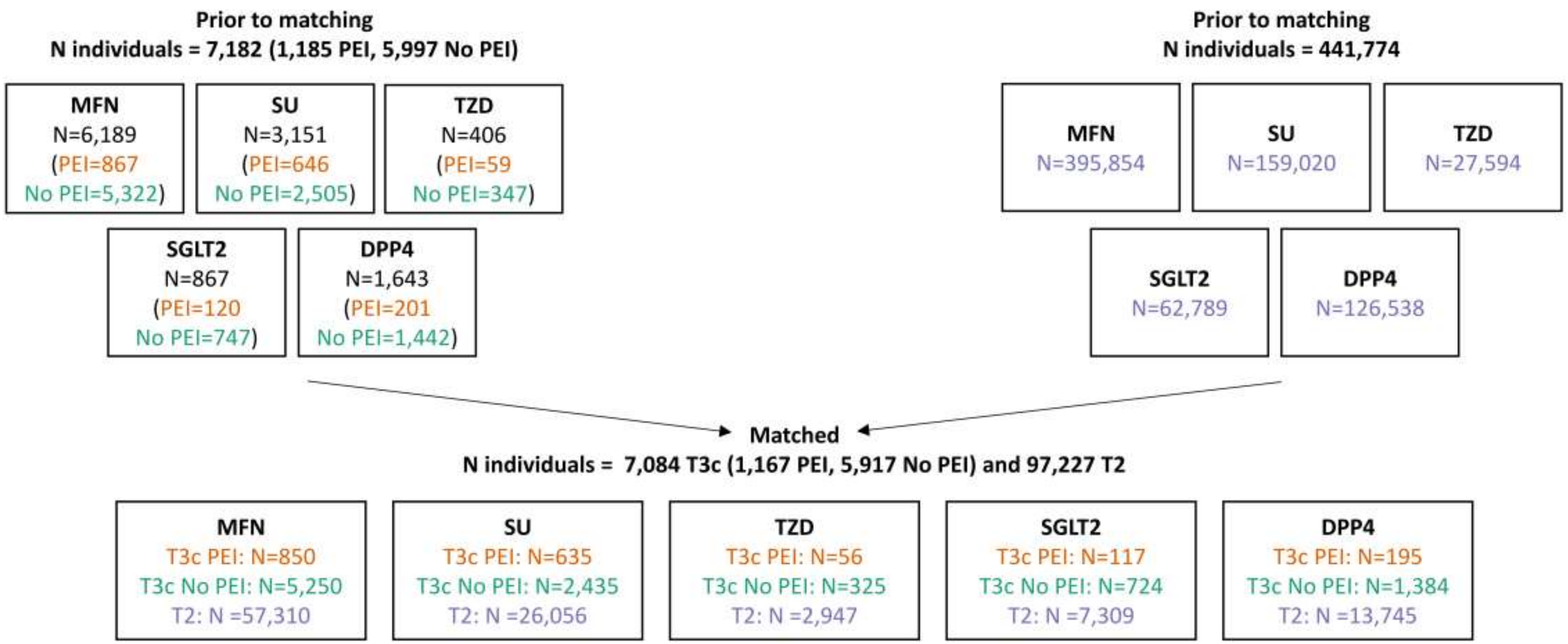

Supplementary Table 1. Definitions of treatment outcomes.

Outcome	Definition
**HbA1c response**	Change from baseline HbA1c 12 months after drug initiation (the closest HbA1c measure to 12 months after initiation within 3-15 months) on unchanged therapy (no addition or cessation of other glucose-lowering medications, and continued prescription of the drug of interest)
**Early treatment discontinuation**	Discontinuation of a therapy within 6 months of initiation, with the availability of at least 3 months follow-up time after discontinuation required to confirm the drug was discontinued. A gap of over 6 months in prescriptions was used to indicate a drug being stopped.
**Weight change**	Change from baseline weight 12 months after drug initiation (the closest weight measure to 12 months after initiation within 3-15 months) on unchanged therapy (no addition or cessation of other glucose-lowering medications, and continued prescription of the drug of interest)

Supplementary Table 2. Definitions of covariates.

Variable	Definition	Data source
**Sex**	Sex recorded in CPRD	Primary care
**Age**	Current age (continuous) in years	Primary care
**Ethnicity**	Major UK ethnic group (White, south Asian, Black, Other, Mixed)	Primary care
**Deprivation**	Index of Multiple Deprivation (official national measure) quintiles (1 (least deprived) – 5 (most deprived))	Linked Index of Multiple Deprivation dataset
**HbA1c**	Baseline HbA1c (closest HbA1c value within 6 months before and 7 days after index date)	Primary care
**BMI**	Baseline BMI (closest value within 2 years before and 7 days after index date)	Primary care
**Weight**	Baseline weight (closest value within 2 years before and 7 days after index date)	Primary care
**Number of other glucose-lowering therapies**	Count of the number of other glucose lowering therapies being taken	Primary care
**Alcohol consumption**	Level of alcohol consumption (none, within government recommended limits, exceeding recommended limits, clinically harmful)	Primary care
**Duration of diabetes**	Current duration of diabetes (continuous) in years	Primary care
**Microvascular complications**	Count of the number of microvascular complications (diabetic nephropathy, retinopathy, neuropathy) prior to the index date (0, 1, 2, or 3)	Primary care or hospital data
**Heart failure**	Record of heart failure prior to index date (yes/no)	Primary care or hospital data
**Atherosclerotic cardiovascular disease**	Record of atherosclerotic cardiovascular disease (myocardial infarction, ischaemic heart disease, stroke, peripheral arterial disease, cardiac revascularisation) prior to index date (yes/no)	Primary care or hospital data
**Chronic kidney disease**	Chronic kidney disease (stages 3-5, identified using eGFR values and medical codes) prior to index date (yes/no)	Primary care or hospital data

Supplementary Figure 3. Kaplan-Meier cumulative incidence curve of time to initiation of insulin within 3 years of diabetes diagnosis for individuals with type 3c diabetes and PEI prior to diabetes diagnosis, type 3c diabetes without PEI prior to diagnosis, and individuals with type 2 diabetes.

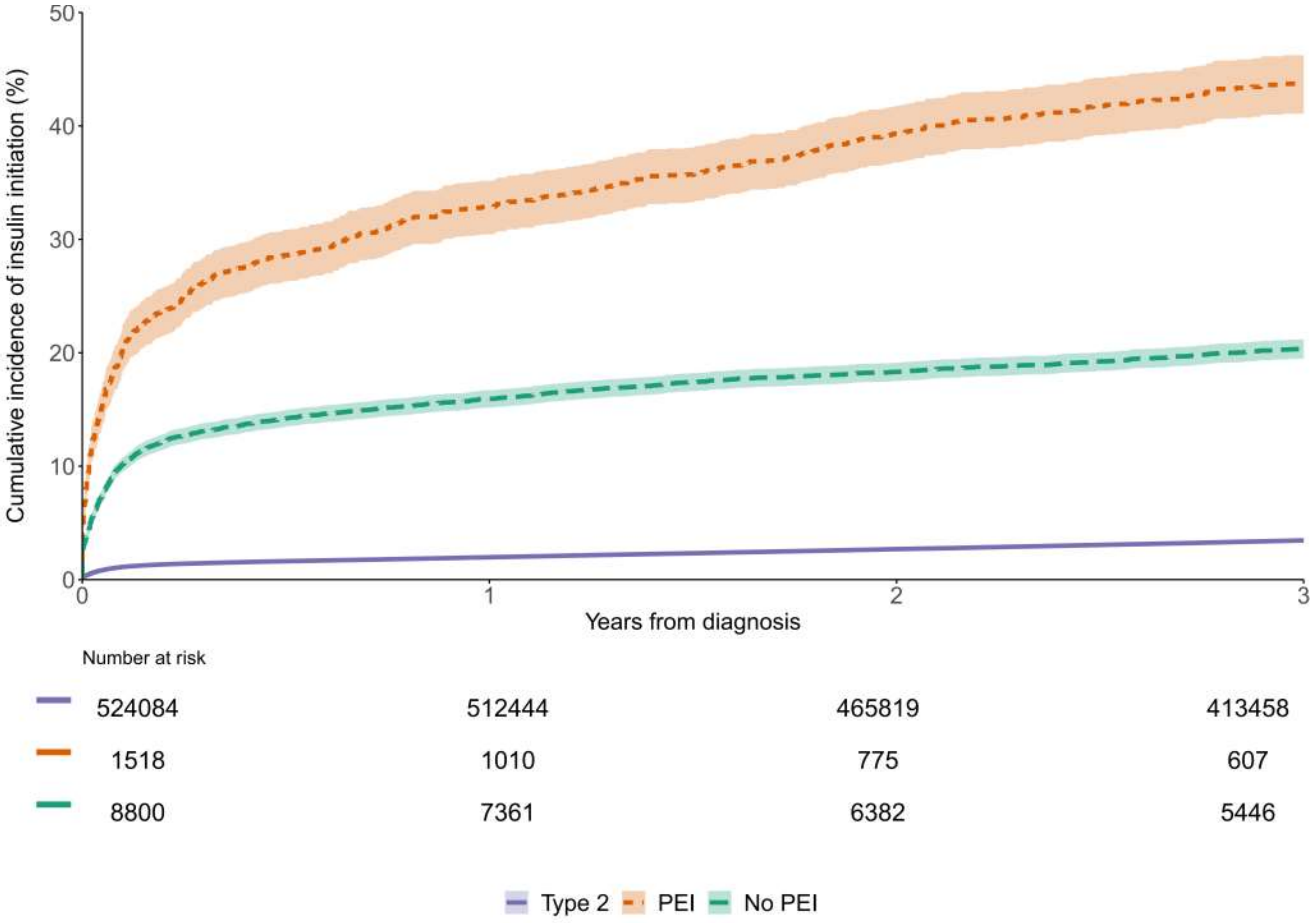

Supplementary Figure 4. Kaplan-Meier cumulative incidence curve of time to initiation of insulin within 3 years of diabetes diagnosis for individuals with type 3c diabetes, stratified by subtype, and individuals with type 2 diabetes.

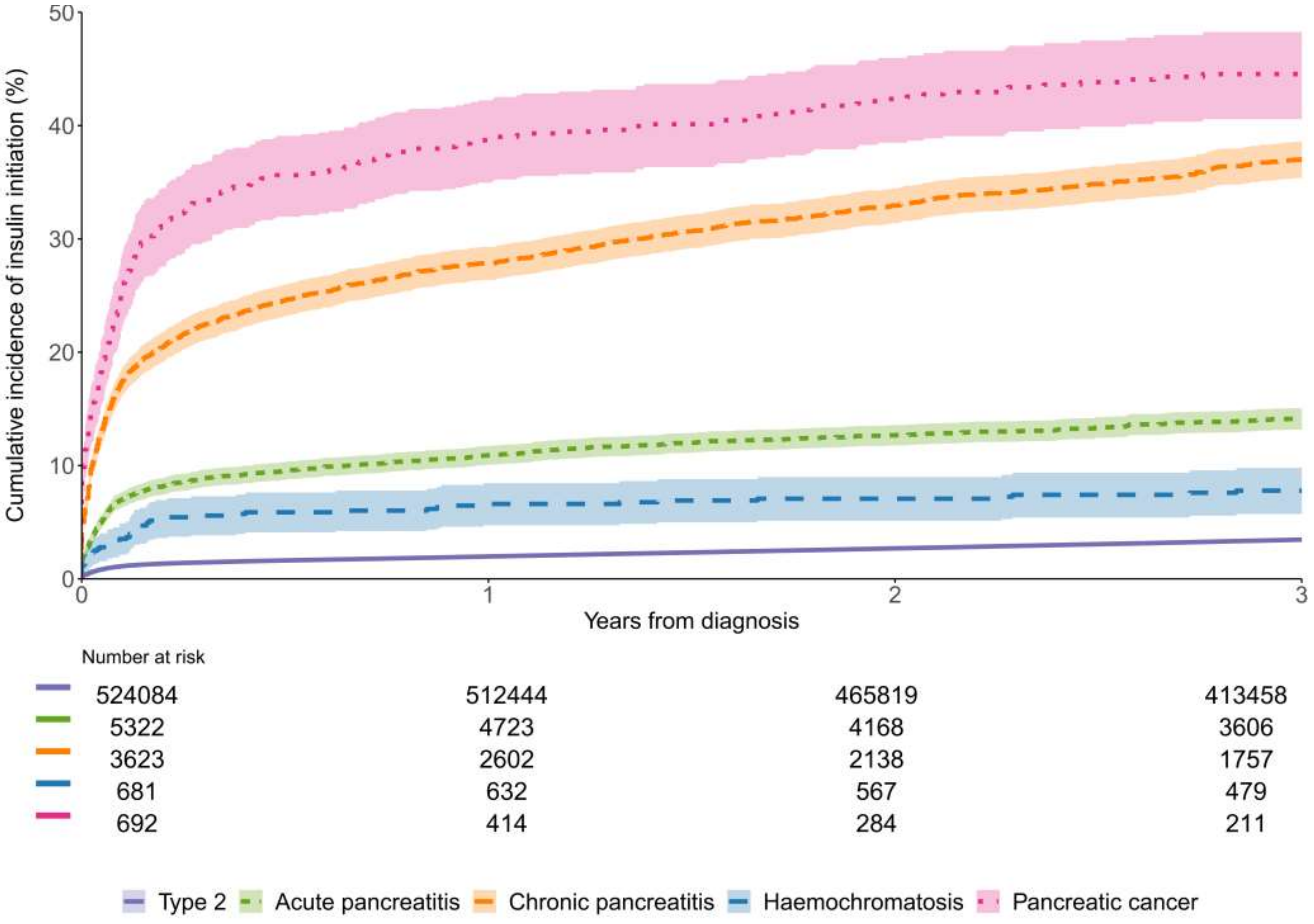

Supplementary Figure 5. Kaplan-Meier cumulative incidence curve of time to initiation of oral glucose-lowering therapy within 3 years of diabetes diagnosis for individuals with type 3c diabetes and PEI prior to diabetes diagnosis, type 3c diabetes without PEI prior to diagnosis, and individuals with type 2 diabetes.

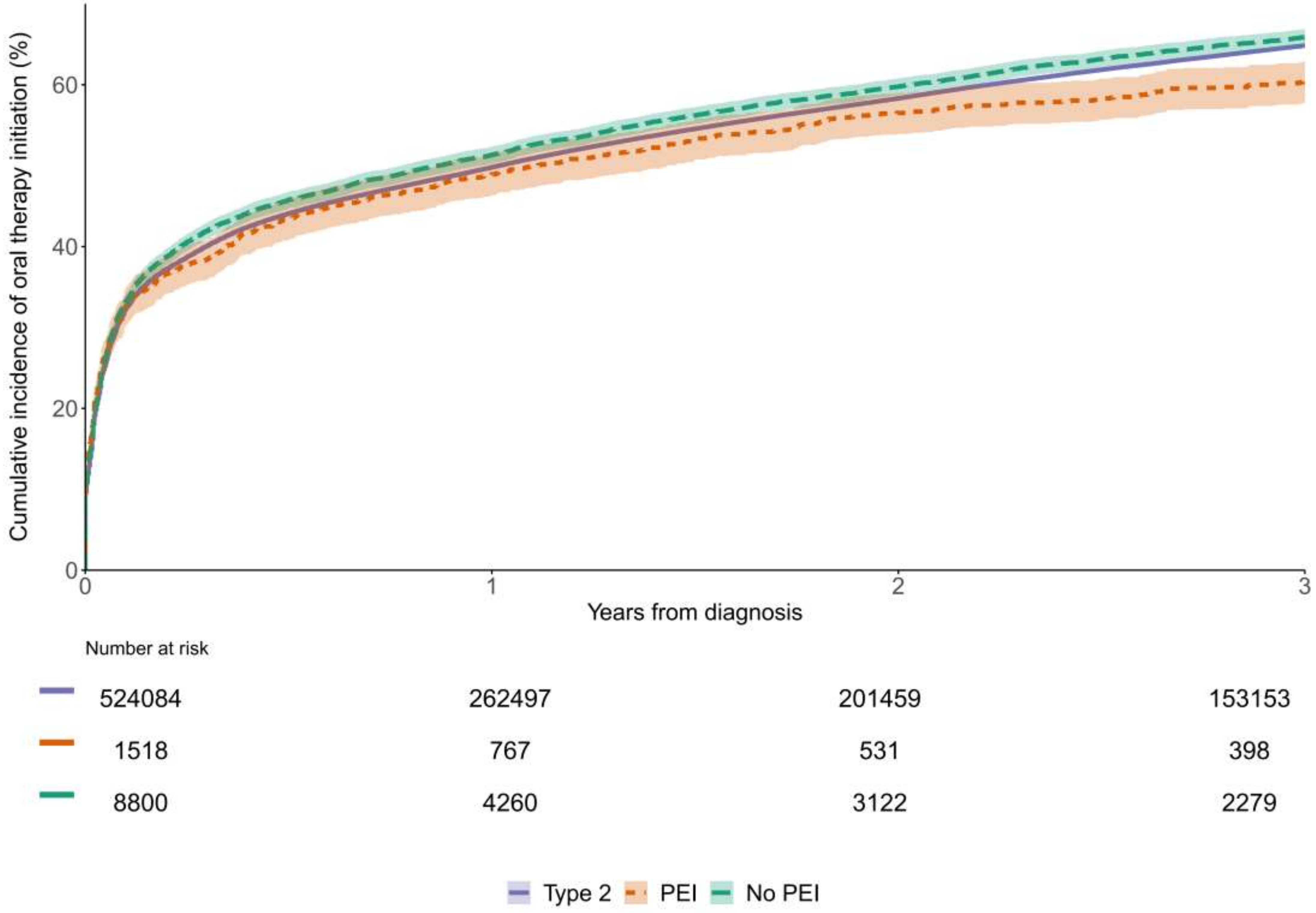

Supplementary Figure 6. Kaplan-Meier cumulative incidence curve of time to initiation of oral glucose-lowering therapy within 3 years of diabetes diagnosis for individuals with type 3c diabetes, stratified by subtype, and individuals with type 2 diabetes.

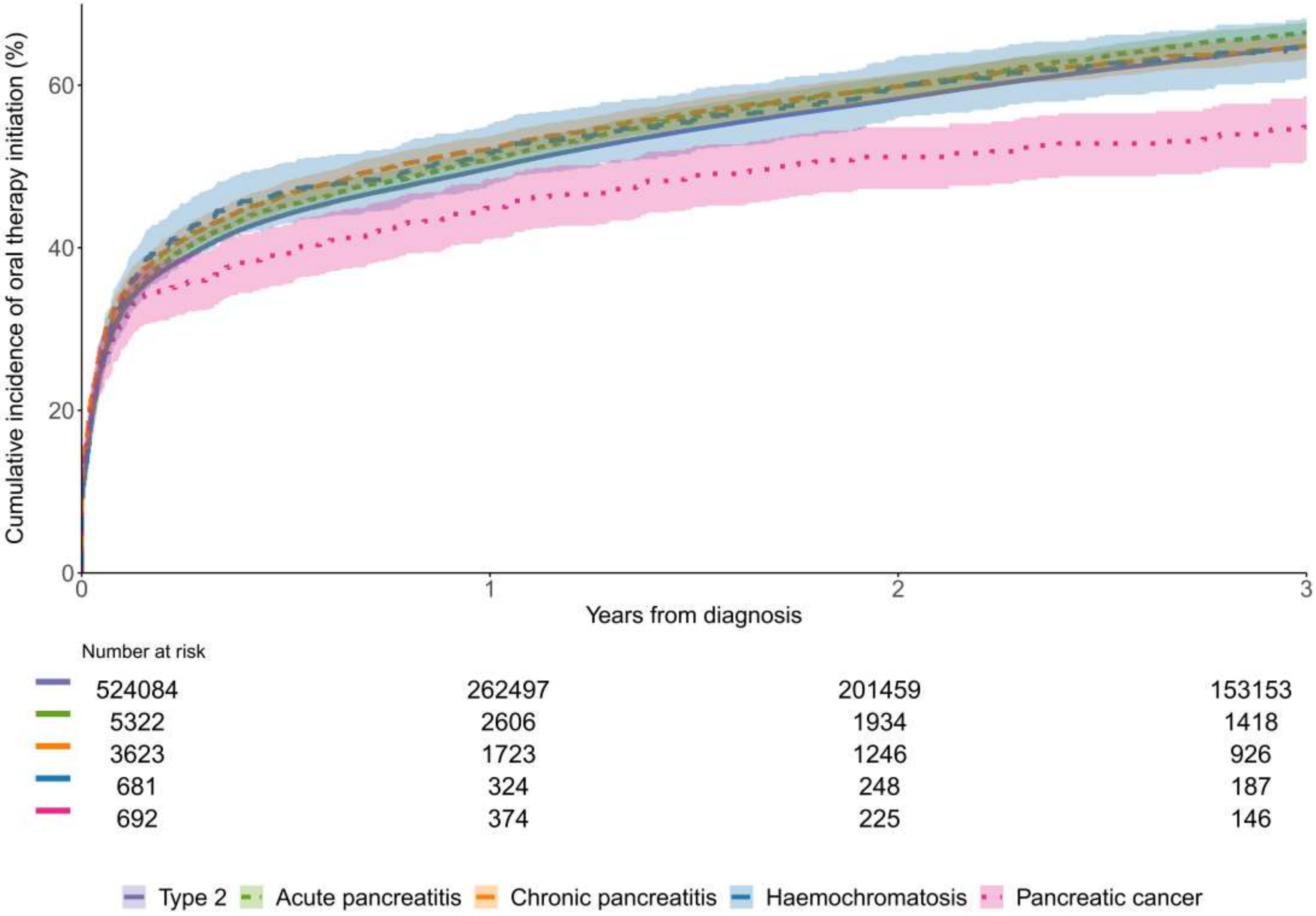

Supplementary Table 3. Baseline characteristics of matched cohort of individuals with type 3c diabetes and type 2 controls initiating a major glucose-lowering therapy class (metformin, sulphonylureas, thiazolidinediones [TZDs], SGLT2-inhibitors, DPP4-inhibitors).

	**Metformin**		**Sulphonylureas**		**TZDs**		**SGLT2-inhibitors**		**DPP4-inhibitors**	
	**T2 controls**	**T3c**	**T2 controls**	**T3c**	**T2 controls**	**T3c**	**T2 controls**	**T3c**	**T2 controls**	**T3c**
n	57310	6100	26056	3070	2947	381	7309	841	13745	1579
**Sex**										
Male	32831 (57.3)	3489 (57.2)	15857 (60.9)	1836 (59.8)	1820 (61.8)	226 (59.3)	4131 (56.5)	461 (54.8)	7901 (57.5)	887 (56.2)
Female	24479 (42.7)	2611 (42.8)	10199 (39.1)	1234 (40.2)	1127 (38.2)	155 (40.7)	3178 (43.5)	380 (45.2)	5844 (42.5)	692 (43.8)
**Age, years**										
Median [IQR]	61.42 [51.95, 70.68]	61.19 [51.68, 70.71]	61.63 [52.66, 70.98]	60.57 [50.92, 70.42]	58.70 [52.99, 65.80]	59.03 [52.53, 66.89]	58.79 [52.32, 66.11]	58.67 [51.39, 66.46]	63.20 [54.40, 71.76]	63.17 [53.80, 72.04]
**Ethnicity**										
White	52799 (92.1)	5510 (90.3)	24890 (95.5)	2836 (92.4)	2877 (97.6)	359 (94.2)	6909 (94.5)	763 (90.7)	12929 (94.1)	1437 (91.0)
South Asian	2938 (5.1)	342 (5.6)	795 (3.1)	150 (4.9)	60 (2.0)	17 (4.5)	306 (4.2)	51 (6.1)	615 (4.5)	98 (6.2)
Black	1112 (1.9)	143 (2.3)	325 (1.2)	62 (2.0)	10 (0.3)	5 (1.3)	66 (0.9)	13 (1.5)	186 (1.4)	36 (2.3)
Other	148 (0.3)	39 (0.6)	7 (0.0)	5 (0.2)	NA	NA	4 (0.1)	3 (0.4)	3 (0.0)	2 (0.1)
Mixed	114 (0.2)	37 (0.6)	19 (0.1)	9 (0.3)	NA	NA	9 (0.1)	7 (0.8)	4 (0.0)	4 (0.3)
Unknown	199 (0.3)	29 (0.5)	20 (0.1)	8 (0.3)	NA	NA	15 (0.2)	4 (0.5)	8 (0.1)	2 (0.1)
**Index of multiple deprivation quintile**										
1 (least deprived)	9915 (17.3)	1045 (17.1)	4389 (16.8)	499 (16.3)	492 (16.7)	66 (17.3)	1306 (17.9)	147 (17.5)	2170 (15.8)	261 (16.5)
2	9701 (16.9)	1025 (16.8)	4487 (17.2)	534 (17.4)	445 (15.1)	62 (16.3)	1284 (17.6)	149 (17.7)	2293 (16.7)	266 (16.8)
3	10962 (19.1)	1169 (19.2)	5013 (19.2)	590 (19.2)	636 (21.6)	76 (19.9)	1427 (19.5)	167 (19.9)	2773 (20.2)	314 (19.9)
4	12246 (21.4)	1304 (21.4)	5623 (21.6)	660 (21.5)	575 (19.5)	79 (20.7)	1419 (19.4)	163 (19.4)	2880 (21.0)	327 (20.7)
5 (most deprived)	14485 (25.3)	1556 (25.5)	6544 (25.1)	787 (25.6)	799 (27.1)	98 (25.7)	1873 (25.6)	215 (25.6)	3629 (26.4)	411 (26.0)
**Alcohol consumption**										
None	3985 (7.0)	498 (8.2)	1769 (6.8)	226 (7.4)	249 (8.4)	35 (9.2)	428 (5.9)	62 (7.4)	938 (6.8)	110 (7.0)
Within limits	44332 (77.4)	4067 (66.7)	20244 (77.7)	1939 (63.2)	2280 (77.4)	256 (67.2)	6190 (84.7)	624 (74.2)	11359 (82.6)	1147 (72.6)
Excess	3841 (6.7)	337 (5.5)	1681 (6.5)	174 (5.7)	213 (7.2)	22 (5.8)	314 (4.3)	40 (4.8)	726 (5.3)	72 (4.6)
Harmful	2484 (4.3)	970 (15.9)	1257 (4.8)	615 (20.0)	108 (3.7)	61 (16.0)	322 (4.4)	108 (12.8)	552 (4.0)	234 (14.8)
Unknown	2668 (4.7)	228 (3.7)	1105 (4.2)	116 (3.8)	97 (3.3)	7 (1.8)	55 (0.8)	7 (0.8)	170 (1.2)	16 (1.0)
**HbA1c, mmol/mol**										
Median [IQR]	61.87 [54.00, 78.14]	62.83 [54.00, 82.00]	74.00 [62.83, 94.33]	75.00 [62.00, 95.00]	71.58 [62.92, 84.00]	72.84 [62.92, 85.79]	73.00 [64.00, 86.00]	73.00 [64.00, 85.00]	68.00 [61.00, 80.00]	69.00 [60.00, 81.00]
**BMI, kg/m2**										
Median [IQR]	32.10 [28.30, 36.90]	30.60 [26.50, 35.50]	31.00 [27.23, 35.50]	28.60 [24.70, 33.30]	32.10 [28.70, 36.40]	29.45 [25.60, 34.69]	33.50 [29.77, 38.20]	32.17 [27.80, 37.30]	32.00 [28.22, 36.50]	30.40 [26.20, 35.00]
**Weight, kg**										
Median [IQR]	92.00 [79.50, 107.00]	87.00 [74.00, 102.00]	89.20 [76.60, 104.00]	81.50 [69.85, 96.00]	93.00 [80.90, 107.00]	83.85 [70.11, 101.75]	97.00 [84.20, 112.00]	92.10 [79.00, 108.00]	91.80 [79.40, 106.00]	86.00 [74.00, 101.00]
**Number of other glucose-lowering therapies being taken**										
0	53940 (94.1)	5530 (90.7)	8742 (33.6)	1271 (41.4)	201 (6.8)	43 (11.3)	558 (7.6)	81 (9.6)	2018 (14.7)	300 (19.0)
1	3275 (5.7)	556 (9.1)	15371 (59.0)	1628 (53.0)	1566 (53.1)	196 (51.4)	3418 (46.8)	415 (49.3)	7913 (57.6)	865 (54.8)

	Metformin		Sulphonylureas		TZDs		SGLT2-inhibitors		DPP4-inhibitors	
	T2 controls	T3c	T2 controls	T3c	T2 controls	T3c	T2 controls	T3c	T2 controls	T3c
2+	95 (0.2)	14 (0.2)	1943 (7.5)	171 (5.6)	1180 (40.0)	142 (37.3)	3333 (45.6)	345 (41.0)	3814 (27.7)	414 (26.2)
**PEI status**										
No PEI	0 (0.0)	5250 (86.1)	0 (0.0)	2435 (79.3)	0 (0.0)	325 (85.3)	0 (0.0)	724 (86.1)	0 (0.0)	1384 (87.7)
PEI	0 (0.0)	850 (13.9)	0 (0.0)	635 (20.7)	0 (0.0)	56 (14.7)	0 (0.0)	117 (13.9)	0 (0.0)	195 (12.3)
**3c subtype**										
Acute pancreatitis only	0 (0.0)	3516 (57.6)	0 (0.0)	1585 (51.6)	0 (0.0)	207 (54.3)	0 (0.0)	515 (61.2)	0 (0.0)	943 (59.7)
Chronic pancreatitis	0 (0.0)	1882 (30.9)	0 (0.0)	1143 (37.2)	0 (0.0)	147 (38.6)	0 (0.0)	239 (28.4)	0 (0.0)	469 (29.7)
Haemochromatosis	0 (0.0)	452 (7.4)	0 (0.0)	171 (5.6)	0 (0.0)	15 (3.9)	0 (0.0)	66 (7.8)	0 (0.0)	115 (7.3)
Pancreatic cancer	0 (0.0)	250 (4.1)	0 (0.0)	171 (5.6)	0 (0.0)	12 (3.1)	0 (0.0)	21 (2.5)	0 (0.0)	52 (3.3)
**Duration of diabetes**										
Median [IQR]	0.39 [0.04, 2.50]	0.25 [0.03, 1.81]	2.47 [0.36, 5.25]	1.39 [0.13, 3.80]	3.19 [1.41, 5.58]	2.74 [1.03, 5.44]	6.98 [4.01, 10.03]	5.07 [2.94, 8.07]	5.72 [3.20, 8.58]	4.28 [2.12, 7.19]
**Number of microvascular complications**										
0	48707 (85.0)	5159 (84.6)	18330 (70.3)	2212 (72.1)	1891 (64.2)	240 (63.0)	3981 (54.5)	504 (59.9)	7365 (53.6)	897 (56.8)
1	7550 (13.2)	842 (13.8)	6343 (24.3)	719 (23.4)	879 (29.8)	114 (29.9)	2608 (35.7)	281 (33.4)	5015 (36.5)	534 (33.8)
2	1031 (1.8)	98 (1.6)	1348 (5.2)	137 (4.5)	173 (5.9)	27 (7.1)	705 (9.6)	54 (6.4)	1319 (9.6)	143 (9.1)
3	22 (0.0)	1 (0.0)	35 (0.1)	2 (0.1)	4 (0.1)	0 (0.0)	15 (0.2)	2 (0.2)	46 (0.3)	5 (0.3)
**Macrovascular complications**										
Heart failure	3223 (5.6)	418 (6.9)	2059 (7.9)	247 (8.0)	82 (2.8)	8 (2.1)	360 (4.9)	49 (5.8)	1040 (7.6)	143 (9.1)
Atherosclerotic cardiovascular disease	12293 (21.5)	1464 (24.0)	6447 (24.7)	765 (24.9)	551 (18.7)	84 (22.0)	1362 (18.6)	181 (21.5)	3434 (25.0)	423 (26.8)
Chronic kidney disease	5853 (10.2)	695 (11.4)	3583 (13.8)	425 (13.8)	275 (9.3)	36 (9.4)	230 (3.1)	38 (4.5)	2014 (14.7)	257 (16.3)

Supplementary Figure 7. A) Mean HbA1c response and B) Proportion of early treatment discontinuation, in individuals with type 3c diabetes following pancreatic cancer with PEI (orange) and without PEI (green), and matched type 2 controls (blue/purple) initiating an oral glucose lowering therapy. Contrasts represent estimated differences between groups with 95% confidence intervals for HbA1c change in mmol/mol and odds ratios with 95% confidence intervals for discontinuation. Models were adjusted for baseline HbA1c and number of other glucose-lowering therapies being taken.

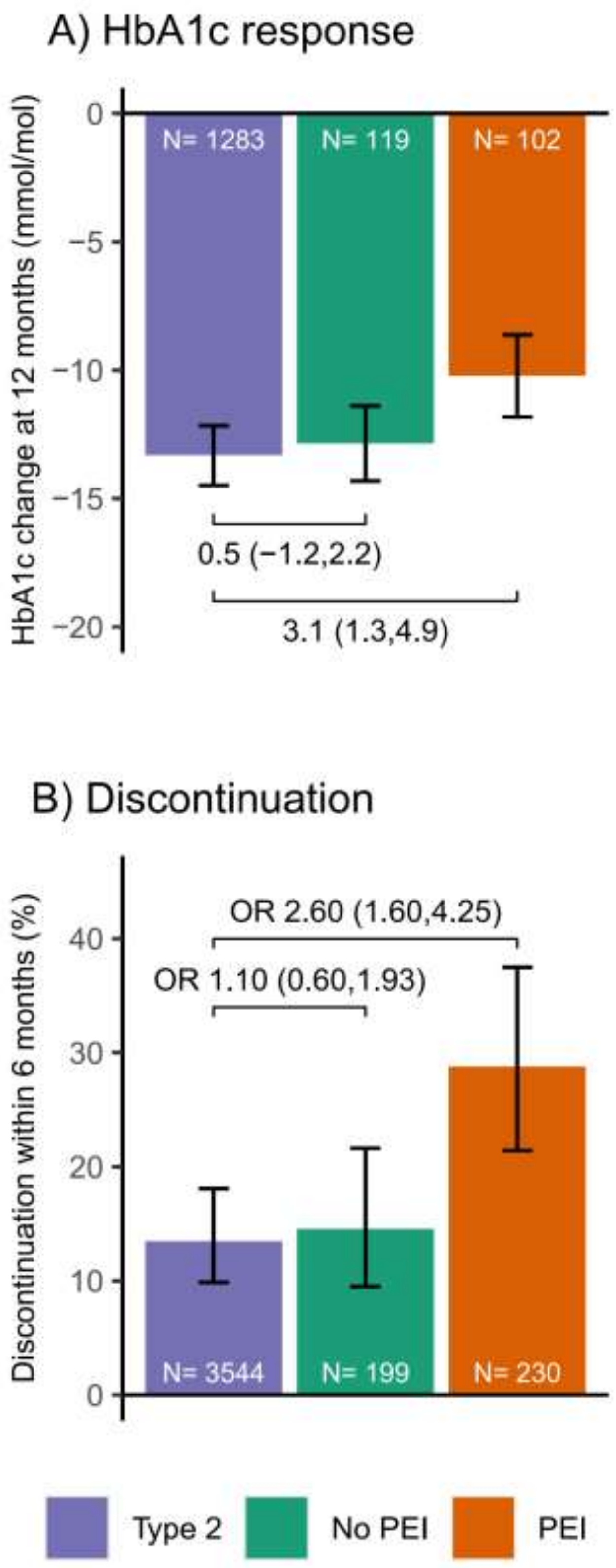

Supplementary Figure 8. A) Mean HbA1c response and B) Proportion of early treatment discontinuation, in individuals with type 3c diabetes following haemochromatosis without PEI (green), and matched type 2 controls (blue/purple) initiating an oral glucose lowering therapy. Contrasts represent estimated differences between groups with 95% confidence intervals for HbA1c change in mmol/mol and odds ratios with 95% confidence intervals for discontinuation. Models were adjusted for baseline HbA1c and number of other glucose-lowering therapies being taken.

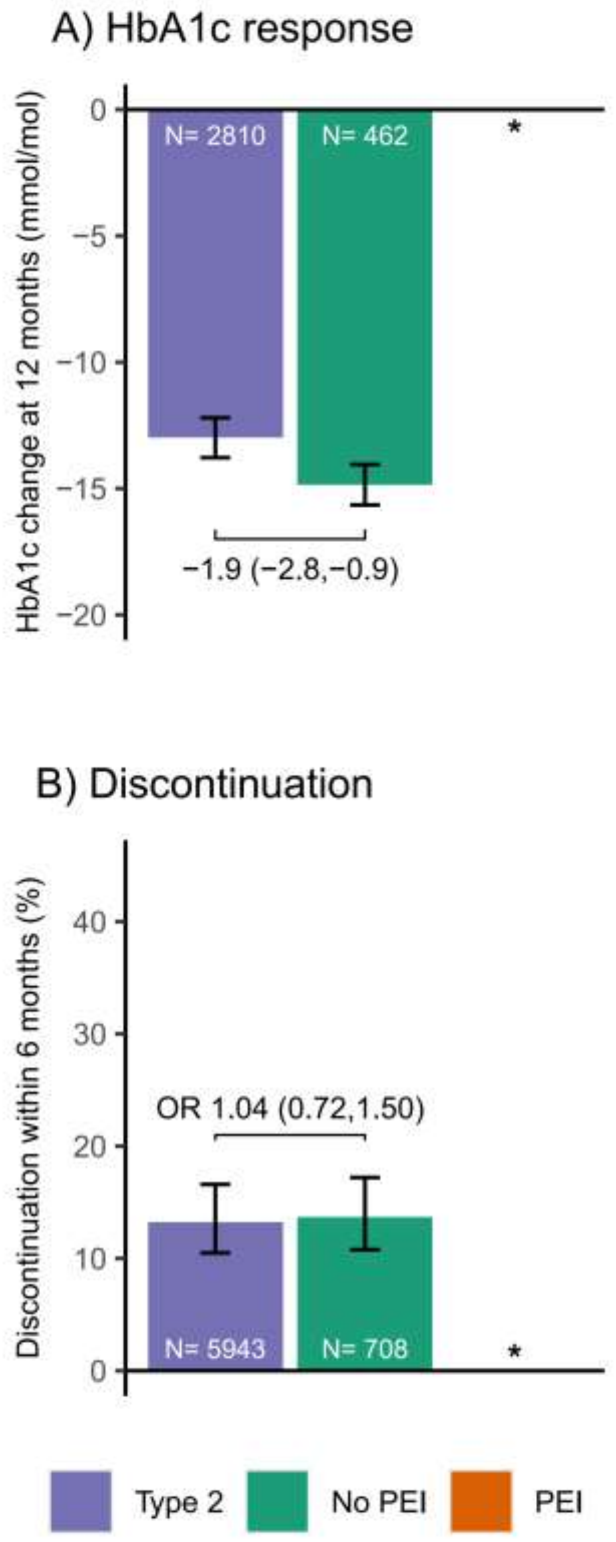

*Due to small numbers of individuals with type 3c diabetes following haemochromatosis with PEI these individuals were not included in this analysis.

Supplementary Table 4. Treatment response outcomes by drug class in all individuals with type 3c diabetes and type 2 controls.

A) **HbA1c**

Drug class	Type 2			Type 3c PEI				Type 3c No PEI			
	N	Unadjusted mean HbA1c change, mmol/mol	Adjusted mean HbA1c change (95% CI), mmol/mol	N	Unadjusted mean HbA1c change, mmol/mol	Adjusted mean HbA1c change (95% CI), mmol/mol	Mean difference vs Type 2 (95% CI), mmol/mol	N	Unadjusted mean HbA1c change, mmol/mol	Adjusted mean HbA1c change (95% CI), mmol/mol	Mean difference vs Type 2 (95% CI), mmol/mol
Metformin	22581	-13.2	-17.4 (-17.6,-17.1)	404	-8.5	-13.1 (-13.8,-12.4)	4.2 (3.5,5.0)	3090	-12.9	-17.2 (-17.4,-16.9)	0.2 (-0.1,0.5)
Sulphonylureas	6398	-16.7	-14.2 (-14.8,-13.7)	226	-11.9	-10.1 (-11.4,-8.8)	4.2 (2.8,5.5)	1095	-15.8	-12.6 (-13.2,-12.0)	1.6 (0.9,2.4)
TZDs	800	-11.1	-12.9 (-14.4,-11.5)	18	-7.7	-10.7 (-14.6,-6.7)	2.3 (-1.9,6.4)	139	-12.6	-12.6 (-14.1,-11.1)	0.3 (-1.6,2.3)
DPP4−inhibitors	4634	-7.3	-8.2 (-8.8,-7.6)	83	-6.1	-6.9 (-8.7,-5.1)	1.3 (-0.6,3.2)	743	-6.4	-7.4 (-8.0,-6.8)	0.8 (0,1.7.0)
SGLT2−inhibitors	1871	-13.4	-11.5 (-12.4,-10.7)	51	-12.8	-12.9 (-15.2,-10.7)	-1.4 (-3.8,1.0)	341	-12.3	-10.5 (-11.4,-9.5)	1.1 (-0.1,2.3)
Overall	36284	-13.0	-12.9 (-13.2,-12.7)	782	-9.5	-9.4 (-10,-8.9)	3.5 (2.9,4.1)	5408	-12.6	-12.2 (-12.4,-12)	0.7 (0.4,1)

B) **Discontinuation**

Drug class	Type 2			Type 3c PEI				Type 3c No PEI			
	N	Unadjusted mean discontinuation, %	Adjusted mean discontinuation (95% CI), %	N	Unadjusted mean discontinuation, %	Adjusted mean discontinuation (95% CI), %	Odds ratio vs Type 2 (95% CI)	N	Unadjusted mean discontinuation, %	Adjusted mean discontinuation (95% CI), %	Odds ratio vs Type 2 (95% CI)
Metformin	45194	5.2	5.1 (4.5,5.8)	726	14.5	13.9 (11.3,16.8)	2.97 (2.29,3.84)	4638	7.0	6.4 (5.6,7.2)	1.26 (1.05,1.50)
Sulphonylureas	21084	17.9	17.8 (16.1,19.5)	546	24.4	24.6 (20.5,29.2)	1.51 (1.17,1.94)	2148	18.7	17.7 (15.8,19.7)	0.99 (0.84,1.17)
TZDs	2536	20.2	19.2 (14.8,24.5)	47	25.5	24.6 (13.1,41.2)	1.37 (0.58,2.99)	299	20.4	19.8 (15.0,25.7)	1.04 (0.68,1.58)
DPP4−inhibitors	11198	16.6	16.0 (14.0,18.2)	165	24.8	23.8 (17.5,31.6)	1.64 (1.07,2.47)	1214	19.0	17.8 (15.6,20.2)	1.14 (0.91,1.41)
SGLT2−inhibitors	5580	20.0	21.4 (18.1,25.2)	94	25.5	27.8 (1.09,38.6)	1.41 (0.81,2.36)	613	19.9	20.6 (17.0,24.6)	0.95 (0.71,1.27)
Overall	85592	11.2	13.5 (12.7,14.3)	1578	20.0	24 (21.5,26.6)	2.03 (1.73,2.36)	8912	12.8	14.4 (13.5,15.3)	1.08 (0.98,1.19)

C) **Weight**

Drug class	Type 2			Type 3c PEI				Type 3c No PEI			
	N	Unadjusted mean weight change, kg	Adjusted mean weight change (95% CI), kg	N	Unadjusted mean weight change, kg	Adjusted mean weight change (95% CI), kg	Mean difference vs Type 2 (95% CI), kg	N	Unadjusted mean weight change, kg	Adjusted mean weight change (95% CI), kg	Mean difference vs Type 2 (95% CI), kg
Metformin	19770	-2.7	-2.7 (-2.8,-2.6)	396	-2.2	-3.2 (-3.5,-2.8)	-0.4 (-0.8,-0.1)	2934	-2.7	-2.9 (-3.0,-2.7)	-0.1 (-0.3,0.0)
Sulphonylureas	7374	1.3	1.3 (1.2,1.5)	267	0.4	0.2 (-0.2,0.6)	-1.1 (-1.5,-0.7)	1191	1.2	1.1 (0.9,1.3)	-0.2 (-0.4,0.0)
TZDs	1031	2.5	2.0 (1.5,2.5)	22	1.4	1.0 (-0.4,2.5)	-1.0 (-2.5,0.5)	171	2.1	1.7 (1.2,2.2)	-0.3 (-1.0,0.4)
DPP4−inhibitors	4297	-1.3	-1.3 (-1.5,-1.1)	88	-1.2	-1.5 (-2.1,-0.9)	-0.2 (-0.8,0.5)	703	-0.8	-0.9 (-1.1,-0.7)	0.4 (0.1,0.7)
SGLT2−inhibitors	1895	-4.1	-3.8 (-4.2,-3.5)	54	-4.7	-5.3 (-6.1,-4.4)	-1.4 (-2.3,-0.6)	351	-4.0	-3.9 (-4.2,-3.5)	0.0 (-0.5,0.4)
Overall	34367	-1.6	-0.8 (-0.9,-0.8)	827	-1.3	-1.4 (-1.6,-1.2)	-0.5 (-0.8,-0.3)	5350	-1.5	-1 (-1.1,-0.9)	-0.1 (-0.3,0)

Supplementary Table 5. Treatment response outcomes by drug class in individuals with type 3c diabetes following acute pancreatitis and type 2 controls.

A) **HbA1c**

Drug class	Type 2			Type 3c PEI				Type 3c No PEI			
	N	Unadjusted mean HbA1c change, mmol/mol	Adjusted mean HbA1c change (95% CI), mmol/mol	N	Unadjusted mean HbA1c change, mmol/mol	Adjusted mean HbA1c change (95% CI), mmol/mol	Mean difference vs Type 2 (95% CI), mmol/mol	N	Unadjusted mean HbA1c change, mmol/mol	Adjusted mean HbA1c change (95% CI), mmol/mol	Mean difference vs Type 2 (95% CI), mmol/mol
Metformin	13667	-13.2	-17.5 (-17.8,-17.2)	51	-8.8	-15.3 (-17.1,-13.4)	2.3 (0.4,4.2)	2053	-12.7	-17.4 (-17.7,-17.0)	0.2 (-0.2,0.6)
Sulphonylureas	3673	-15.4	-14.2 (-14.8,-13.5)	29	-8.5	-10.3 (-13.5,-7.0)	3.9 (0.6,7.2)	713	-15.2	-13.0 (-13.7,-12.3)	1.1 (0.2,2.1)
TZDs*	445	-11.7	-13.2 (-15.3,-11.1)					88	-12.1	-13.0 (-15.0,-11.0)	0.2 (-2.5,2.9)
DPP4–inhibitors	2878	-7.4	-8.5 (-9.2,-7.7)	18	-6.5	-5.6 (-9.3,-1.8)	2.9 (-1.0,6.7)	494	-5.8	-6.8 (-7.5,-6.1)	1.7 (0.6,2.7)
SGLT2–inhibitors	1166	-13.3	-11.0 (-12.2,-9.9)	17	-20.5	-16.5 (-20.5,-12.4)	-5.5 (-9.6,-1.3)	229	-11.8	-9.8 (-11.0,-8.6)	1.2 (-0.3,2.7)
Overall	21829	-12.8	-13 (-13.3,-12.7)	117	-10.2	-10.9 (-12.3,-9.5)	2 (0.6,3.5)	3577	-12.2	-12.3 (-12.6,-12)	0.7 (0.3,1.1)

B) **Discontinuation**

Drug class	Type 2			Type 3c PEI				Type 3c No PEI			
	N	Unadjusted mean discontinuation, %	Adjusted mean discontinuation (95% CI), %	N	Unadjusted mean discontinuation, %	Adjusted mean discontinuation (95% CI), %	Odds ratio vs Type 2 (95% CI)	N	Unadjusted mean discontinuation, %	Adjusted mean discontinuation (95% CI), %	Odds ratio vs Type 2 (95% CI)
Metformin	26180	5.4	5.2 (4.4,6.1)	85	8.2	7.6 (3.4,16.1)	1.49 (0.56,3.27)	3009	6.4	5.8 (4.9,6.8)	1.12 (0.89,1.42)
Sulphonylureas	11016	17.2	16.9 (14.8,19.3)	57	17.5	20.4 (10.9,34.8)	1.26 (0.56,2.55)	1341	17.7	17.0 (14.8,19.5)	1.01 (0.81,1.26)
TZDs*	1390	19.8	18.5 (13.0,25.7)					187	19.3	19.4 (13.7,26.7)	1.06 (0.61,1.85)
DPP4–inhibitors	6589	16.5	15.7 (13.2,18.5)	30	10.0	10.8 (3.5,28.8)	0.65 (0.15,1.91)	786	19.6	18.4 (15.6,21.4)	1.21 (0.92,1.59)
SGLT2–inhibitors	3450	20.2	21.3 (17.2,26.1)	26	15.4	16.3 (6.2,36.4)	0.72 (0.21,1.96)	417	18.7	18.4 (14.4,23.3)	0.83 (0.58,1.20)
Overall	48625	11.0	13.3 (12.3,14.4)	202	12.4	15 (10.1,21.6)	1.15 (0.71,1.78)	5740	12.2	14 (13,15.2)	1.06 (0.94,1.21)

C) **Weight**

Drug class	Type 2			Type 3c PEI				Type 3c No PEI			
	N	Unadjusted mean weight change, kg	Adjusted mean weight change (95% CI), kg	N	Unadjusted mean weight change, kg	Adjusted mean weight change (95% CI), kg	Mean difference vs Type 2 (95% CI), kg	N	Unadjusted mean weight change, kg	Adjusted mean weight change (95% CI), kg	Mean difference vs Type 2 (95% CI), kg
Metformin	11944	-2.8	-2.9 (-3.0,-2.7)	49	-2.4	-3.2 (-4.1,-2.3)	-0.3 (-1.2,0.6)	1956	-2.7	-2.8 (-2.9,-2.6)	0.1 (-0.1,0.3)
Sulphonylureas	4115	1.1	1.2 (0.9,1.4)	36	-0.2	-0.2 (-1.2,0.8)	-1.4 (-2.4,-0.4)	760	1.3	1.3 (1.1,1.6)	0.2 (-0.1,0.5)
TZDs*	598	2.2	1.7 (1,2.4.0)					109	1.7	1.2 (0.5,2.0)	-0.5 (-1.4,0.5)
DPP4–inhibitors	2621	-1.2	-1.3 (-1.5,-1.0)	20	-1.1	-1.4 (-2.7,-0.1)	-0.1 (-1.4,1.2)	453	-0.8	-0.8 (-1.1,-0.6)	0.4 (0.1,0.8)
SGLT2–inhibitors	1208	-4.1	-3.9 (-4.3,-3.4)	15	-4.3	-5.1 (-6.6,-3.5)	-1.2 (-2.8,0.4)	245	-4.2	-4.0 (-4.4,-3.6)	-0.2 (-0.7,0.4)
Overall	20486	-1.7	-1 (-1.1,-0.9)	123	-1.6	-1.7 (-2.3,-1.1)	-0.7 (-1.3,-0.1)	3523	-1.5	-0.9 (-1,-0.8)	0.1 (0,0.3)

*****No results shown for T3c PEI where N <10 for this group.

Supplementary Table 6. Treatment response outcomes by drug class in individuals with type 3c diabetes following chronic pancreatitis and type 2 controls.

A) HbA1c

Drug class	Type 2			Type 3c PEI				Type 3c No PEI			
	N	Unadjusted mean HbA1c change, mmol/mol	Adjusted mean HbA1c change (95% CI), mmol/mol	N	Unadjusted mean HbA1c change, mmol/mol	Adjusted mean HbA1c change (95% CI), mmol/mol	Mean difference vs Type 2 (95% CI), mmol/mol	N	Unadjusted mean HbA1c change, mmol/mol	Adjusted mean HbA1c change (95% CI), mmol/mol	Mean difference vs Type 2 (95% CI), mmol/mol
Metformin	6297	-13.3	-16.9 (-17.4,-16.4)	304	-8.5	-12.7 (-13.6,-11.8)	4.2 (3.2,5.2)	684	-12.5	-15.6 (-16.2,-15.0)	1.3 (0.5,2.1)
Sulphonylureas	2028	-18.4	-14.2 (-15.2,-13.1)	158	-12.5	-9.5 (-11.2,-7.7)	4.7 (2.8,6.7)	279	-16.2	-10.6 (-11.9,-9.3)	3.6 (2,5.2.0)
TZDs	289	-10.2	-12.1 (-14.3,-10.0)	15	-7.3	-9.9 (-13.8,-5.9)	2.2 (-2.1,6.6)	41	-12.3	-11.5 (-14.2,-8.9)	0.6 (-2.6,3.8)
DPP4−inhibitors	1209	-7.3	-8.0 (-9.2,-6.8)	55	-6.0	-7.2 (-9.5,-4.9)	0.7 (-1.8,3.3)	167	-6.9	-8.0 (-9.4,-6.7)	-0.1 (-1.8,1.7)
SGLT2−inhibitors	529	-14.0	-12.8 (-14.4,-11.3)	29	-7.6	-11.7 (-14.5,-8.9)	1.2 (-1.9,4.3)	79	-12.1	-10.8 (-12.5,-9.0)	2.1 (-0.1,4.2)
Overall	10352	-13.6	-12.8 (-13.2,-12.3)	561	-9.3	-9 (-9.7,-8.2)	3.8 (3,4.6)	1250	-12.5	-10.9 (-11.4,-10.4)	1.8 (1.2,2.5)

B) Discontinuation

Drug class	Type 2			Type 3c PEI				Type 3c No PEI			
	N	Unadjusted mean discontinuation, %	Adjusted mean discontinuation (95% CI), %	N	Unadjusted mean discontinuation, %	Adjusted mean discontinuation (95% CI), %	Odds ratio vs Type 2 (95% CI)	N	Unadjusted mean discontinuation, %	Adjusted mean discontinuation (95% CI), %	Odds ratio vs Type 2 (95% CI)
Metformin	13831	5.1	5.0 (4.0,6.3)	525	14.3	13.9 (11.0,17.4)	3.06 (2.15,4.35)	1135	9.0	8.3 (6.7,10.3)	1.72 (1.24,2.38)
Sulphonylureas	7804	18.6	18.8 (16.1,21.9)	406	25.6	25.3 (20.4,30.8)	1.46 (1.06,1.99)	608	20.7	18.8 (15.3,22.9)	1.00 (0.74,1.34)
TZDs	938	21.1	21.6 (14.3,31.3)	39	28.2	28.5 (14.8,47.8)	1.44 (0.54,3.64)	90	22.2	19.1 (10.9,31.3)	0.86 (0.40,1.80)
DPP4−inhibitors	3336	16.8	16.1 (12.6,20.4)	113	27.4	25.0 (17.4,34.5)	1.74 (1.01,2.93)	299	19.4	18.5 (14.1,23.9)	1.18 (0.77,1.80)
SGLT2−inhibitors	1543	20.2	21.6 (15.6,29.2)	60	28.3	32.1 (20.3,46.7)	1.72 (0.82,3.49)	133	19.5	21.0 (14.0,30.1)	0.96 (0.53,1.72)
Overall	27452	11.7	13.8 (12.5,15.2)	1143	20.8	24.1 (21.2,27.3)	1.98 (1.63,2.41)	2265	14.7	15.7 (13.9,17.6)	1.16 (0.98,1.39)

C) Weight

Drug class	Type 2			Type 3c PEI				Type 3c No PEI			
	N	Unadjusted mean weight change, kg	Adjusted mean weight change (95% CI), kg	N	Unadjusted mean weight change, kg	Adjusted mean weight change (95% CI), kg	Mean difference vs Type 2 (95% CI), kg	N	Unadjusted mean weight change, kg	Adjusted mean weight change (95% CI), kg	Mean difference vs Type 2 (95% CI), kg
Metformin	5476	-2.5	-2.5 (-2.7,-2.3)	292	-1.9	-3.0 (-3.4,-2.6)	-0.5 (-0.9,0.0)	649	-2.6	-3.2 (-3.4,-2.9)	-0.6 (-1.0,-0.3)
Sulphonylureas	2493	1.5	1.5 (1.3,1.8)	195	0.8	0.2 (-0.3,0.8)	-1.3 (-1.9,-0.7)	312	1.0	0.7 (0.3,1.0)	-0.9 (-1.3,-0.4)
TZDs	354	3.3	2.7 (1.9,3.4)	18	1.0	1.6 (0.2,3.0)	-1.1 (-2.6,0.5)	50	2.7	3.0 (2.1,3.9)	0.3 (-0.8,1.4)
DPP4−inhibitors	1194	-1.5	-1.5 (-1.8,-1.1)	56	-1.1	-1.4 (-2.1,-0.7)	0.0 (-0.7,0.8)	174	-0.7	-0.9 (-1.3,-0.5)	0.6 (0,1.1.0)
SGLT2−inhibitors	517	-4.1	-4.0 (-4.7,-3.3)	37	-4.8	-4.9 (-5.9,-3.9)	-0.9 (-2.0,0.3)	72	-3.2	-3.2 (-3.9,-2.5)	0.8 (-0.1,1.7)
Overall	10034	-1.3	-0.5 (-0.7,-0.4)	598	-1.0	-1.2 (-1.5,-1)	-0.7 (-1,-0.4)	1257	-1.3	-1.1 (-1.3,-0.9)	-0.6 (-0.8,-0.4)

Supplementary Table 7. Treatment response outcomes by drug class in individuals with type 3c diabetes following pancreatic cancer and type 2 controls.

A) HbA1c

Drug class	Type 2			Type 3c PEI				Type 3c No PEI			
	N	Unadjusted mean HbA1c change, mmol/mol	Adjusted mean HbA1c change (95% CI), mmol/mol	N	Unadjusted mean HbA1c change, mmol/mol	Adjusted mean HbA1c change (95% CI), mmol/mol	Mean difference vs Type 2 (95% CI), mmol/mol	N	Unadjusted mean HbA1c change, mmol/mol	Adjusted mean HbA1c change (95% CI), mmol/mol	Mean difference vs Type 2 (95% CI), mmol/mol
Metformin	754	-12.9	-18.1 (-19.4,-16.9)	49	-8.5	-14.9 (-16.8,-12.9)	3.2 (1,5.5)	68	-18.0	-17.3 (-18.9,-15.7)	0.8 (-1.2,2.9)
Sulphonylureas	302	-19.6	-14.2 (-16.9,-11.6)	38	-12.3	-9 (-12.6,-5.5)	5.2 (1.4,9)	26	-20.8	-13.4 (-17.2,-9.5)	0.9 (-3.4,5.1)
TZDs*											
DPP4–inhibitors**	172	-6.0	-6.4 (-9.2,-3.6)					18	-12.3	-10.6 (-14.0,-7.2)	-4.2 (-8.5,0.1)
SGLT2–inhibitors*											
Overall	1283	-13.5	-13.3 (-14.5,-12.2)	102	-10.0	-10.2 (-11.8,-8.6)	3.1 (1.3,4.9)	119	-17.2	-12.8 (-14.3,-11.4)	0.5 (-1.2,2.2)

B) Discontinuation

Drug class	Type 2			Type 3c PEI				Type 3c No PEI			
	N	Unadjusted mean discontinuation, %	Adjusted mean discontinuation (95% CI), %	N	Unadjusted mean discontinuation, %	Adjusted mean discontinuation (95% CI), %	Odds ratio vs Type 2 (95% CI)	N	Unadjusted mean discontinuation, %	Adjusted mean discontinuation (95% CI), %	Odds ratio vs Type 2 (95% CI)
Metformin	1808	5.6	5.7 (3.1,10.1)	115	20.0	19.7 (12.4,29.9)	4.09 (1.86,9.38)	99	13.1	12.3 (6.7,21.3)	2.33 (0.95,5.72)
Sulphonylureas	1120	17.4	19.9 (12.4,30.5)	82	23.2	28.3 (16.1,44.7)	1.59 (0.69,3.59)	56	10.7	8.0 (2.6,22.5)	0.35 (0.08,1.14)
TZDs*											
DPP4–inhibitors	394	14.7	13.3 (6.0,27.0)	21	33.3	40.2 (18.8,66.1)	4.38 (1.11,18.06)	27	7.4	4.7 (0.6,26.9)	0.32 (0.02,2.07)
SGLT2–inhibitors**	150	16.7	20.8 (6.5,50.0)					10	40.0	49.0 (15.8,83.1)	3.65 (0.47,33.34)
Overall	3544	11.1	13.5 (9.9,18.1)	230	22.6	28.8 (21.4,37.5)	2.6 (1.6,4.25)	199	13.6	14.6 (9.5,21.6)	1.1 (0.6,1.93)

C) Weight

Drug class	Type 2			Type 3c PEI				Type 3c No PEI			
	N	Unadjusted mean weight change, kg	Adjusted mean weight change (95% CI), kg	N	Unadjusted mean weight change, kg	Adjusted mean weight change (95% CI), kg	Mean difference vs Type 2 (95% CI), kg	N	Unadjusted mean weight change, kg	Adjusted mean weight change (95% CI), kg	Mean difference vs Type 2 (95% CI), kg
Metformin	696	-2.8	-3.0 (-3.6,-2.5)	54	-3.8	-4.7 (-5.5,-3.9)	-1.7 (-2.6,-0.7)	59	-4.7	-5.3 (-6.1,-4.6)	-2.3 (-3.2,-1.4)
Sulphonylureas	324	1.6	1.6 (0.6,2.5)	36	-1.4	-0.6 (-2.1,0.8)	-2.2 (-3.7,-0.6)	30	0.3	1.0 (-0.5,2.5)	-0.5 (-2.2,1.1)
TZDs*											
DPP4–inhibitors	186	-1.6	-1.2 (-2.2,-0.2)	11	-1.2	-1.5 (-3.3,0.2)	-0.3 (-2.3,1.7)	20	-0.1	-0.1 (-1.3,1.2)	1.1 (-0.5,2.7)
SGLT2–inhibitors*											
Overall	1261	-1.4	-0.7 (-1.2,-0.2)	104	-2.7	-2.3 (-3.1,-1.6)	-1.6 (-2.4,-0.8)	117	-2.4	-2.2 (-2.8,-1.5)	-1.4 (-2.2,-0.7)

*No results shown for drug classes where T3c PEI and T3c No PEI groups both have N <10.

**No results shown for T3c PEI where N <10 for this group.

Supplementary Table 8. Treatment response outcomes by drug class in individuals with type 3c diabetes following haemochromatosis and type 2 controls.

A) HbA1c

Drug class	Type 2			Type 3c No PEI			
	N	Unadjusted mean HbA1c change, mmol/mol	Adjusted mean HbA1c change (95% CI), mmol/mol	N	Unadjusted mean HbA1c change, mmol/mol	Adjusted mean HbA1c change (95% CI), mmol/mol	Mean difference vs Type 2 (95% CI)
Metformin	1863	-13.2	-17.4 (-18.2,-16.6)	285	-14.5	-19.2 (-20.0,-18.4)	-1.8 (-2.8,-0.7)
Sulphonylureas	390	-17.1	-13.5 (-15.6,-11.3)	77	-18.4	-15.9 (-18.1,-13.7)	-2.5 (-5.3,0.4)
TZDs*							
DPP4–inhibitors	370	-7.2	-8.2 (-10.5,-5.9)	64	-8.0	-9.4 (-11.7,-7.2)	-1.3 (-4.4,1.8)
SGLT2–inhibitors	136	-12.2	-9.7 (-12.7,-6.7)	28	-17.6	-13.7 (-16.9,-10.5)	-4.0 (-7.8,-0.2)
Overall	2810	-12.9	-13 (-13.8,-12.2)	462	-14.5	-14.8 (-15.6,-14)	-1.9 (-2.8,-0.9)

B) Discontinuation

Drug class	Type 2			Type 3c No PEI			
	N	Unadjusted mean discontinuation, %	Adjusted mean discontinuation (95% CI), %	N	Unadjusted mean discontinuation, %	Adjusted mean discontinuation (95% CI), %	Odds ratio vs Type 2 (95% CI)
Metformin	3367	4.4	4.7 (2.9,7.7)	395	3.8	3.5 (2.0,6.2)	0.73 (0.34,1.55)
Sulphonylureas	1134	20.4	19.6 (12.9,28.6)	143	23.1	22.8 (15.3,32.6)	1.21 (0.64,2.31)
TZDs	136	16.9	8.1 (1.2,39.8)	15	20.0	18.9 (4.2,55.2)	2.65 (0.29,35.65)
DPP4–inhibitors	869	17.8	18.9 (12.0,28.6)	102	16.7	13.4 (7.8,22.2)	0.66 (0.29,1.49)
SGLT2–inhibitors	437	19.0	21.0 (10.6,37.2)	53	26.4	32.1 (18.4,49.8)	1.78 (0.68,4.91)
Overall	5943	10.8	13.2 (10.5,16.6)	708	11.6	13.7 (10.8,17.2)	1.04 (0.72,1.5)

C) Weight

Drug class	Type 2			Type 3c No PEI			
	N	Unadjusted mean weight change, kg	Adjusted mean weight change (95% CI), kg	N	Unadjusted mean weight change, kg	Adjusted mean weight change (95% CI), kg	Mean difference vs Type 2 (95% CI)
Metformin	1648	-2.4	-2.3 (-2.7,-1.9)	270	-2.5	-2.5 (-2.9,-2.1)	-0.2 (-0.7,0.4)
Sulphonylureas	442	1.4	1.9 (1.0,2.9)	89	0.6	1.0 (0.0,1.9)	-1.0 (-2.2,0.2)
TZDs*							
DPP4–inhibitors	290	-1.5	-1.1 (-1.9,-0.2)	56	-1.4	-1.4 (-2.2,-0.5)	-0.3 (-1.4,0.9)
SGLT2–inhibitors	139	-4.2	-3.4 (-4.8,-1.9)	29	-3.8	-4.0 (-5.6,-2.5)	-0.7 (-2.4,1.1)
Overall	2574	-1.6	-0.6 (-1,-0.2)	453	-1.7	-1 (-1.4,-0.7)	-0.4 (-0.9,0)

*No results shown for T3c No PEI group where N <10 for this group.

Supplementary Figure 9. Sensitivity analysis restricted to individuals whose alcohol consumption was within recommended limits or who did not consume alcohol.

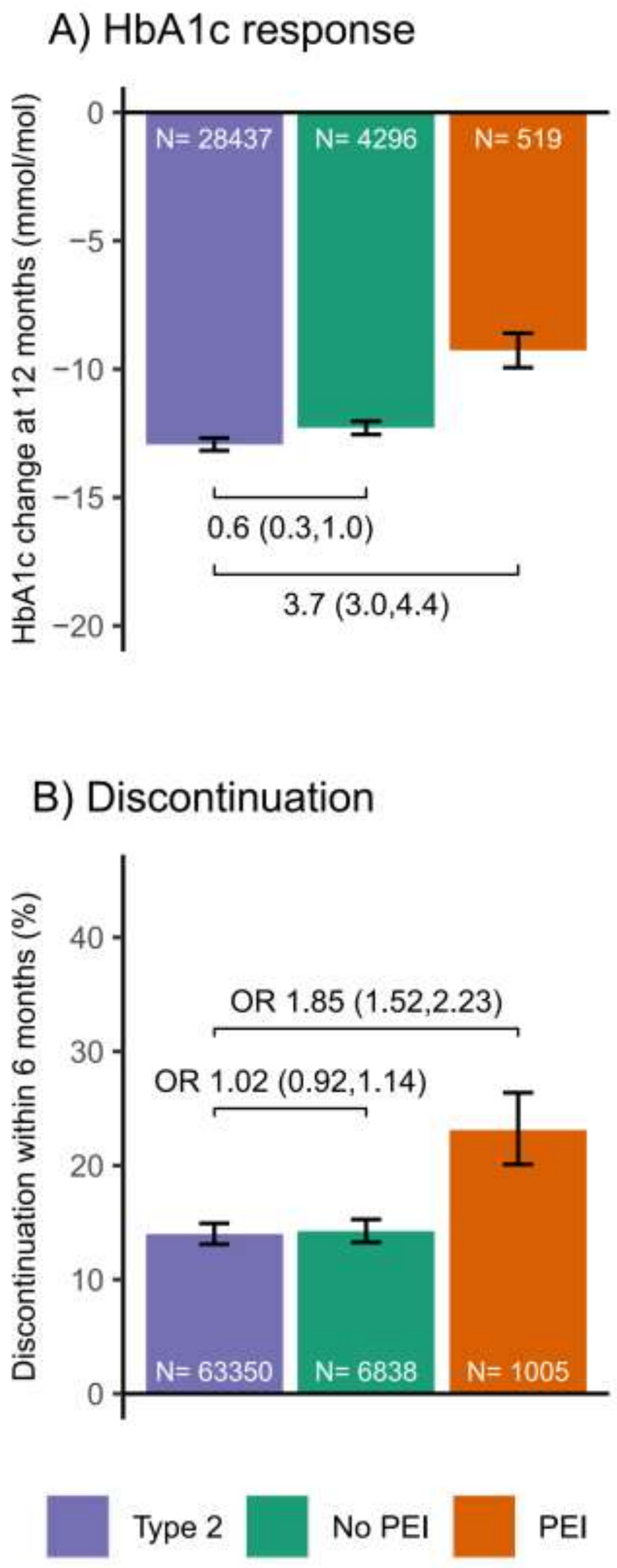

Supplementary Figure 10. Sensitivity analysis restricting the criteria of the PEI subgroup to those with a PERT prescription within the 6 months prior to drug initiation.

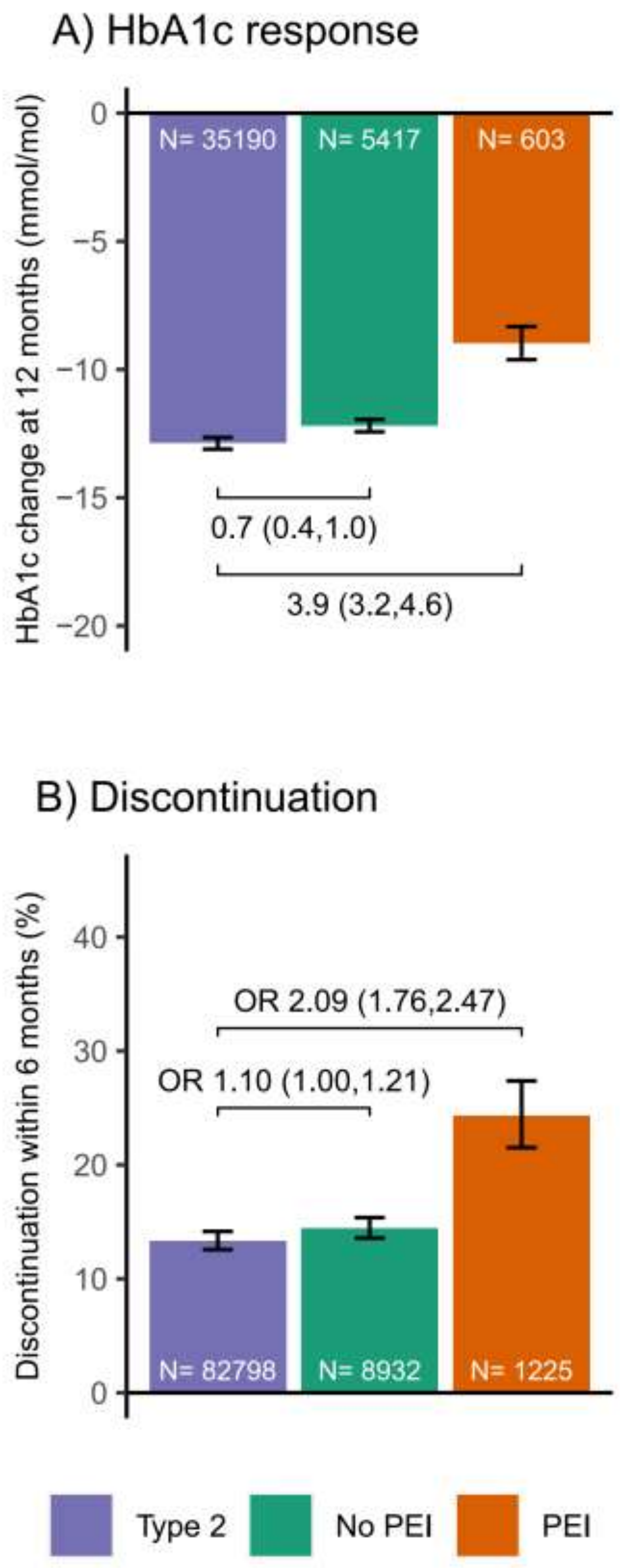

Supplementary Figure 11. Sensitivity analysis restricting the criteria of diabetes following acute pancreatitis to those with a record of acute pancreatitis within the 5 years prior to diabetes diagnosis.

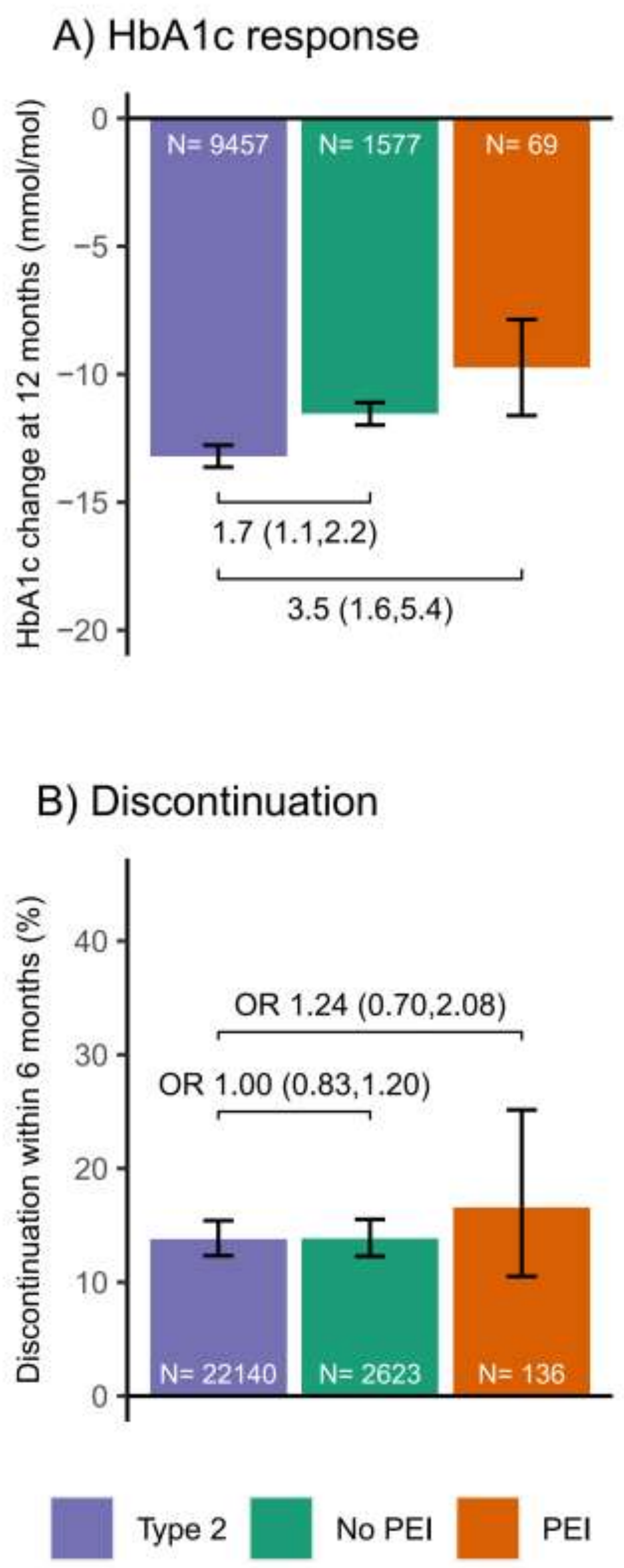

Supplement: Supplementary file 1 — Data S1: [file DOM-27-1544-s002.pdf]
